# Comparative aptamer profiling reveals cell surface remodeling and the emergence of a noncanonical cell surface protein under oncogenic signaling

**DOI:** 10.1039/d5cb00110b

**Published:** 2025-10-03

**Authors:** Jungo Kakuta, Kenji Ohba, Hideaki Ogasawara, Kyohei Okahara, Kazumi Emoto, Hiroaki Sako, Miho Sekai, Yasuyuki Fujita, Toshio Imai, Yogo Sakakibara

**Affiliations:** a KAN Research Institute, Inc. Kobe Hyogo Japan y3-sakakibara@hhc.eisai.co.jp; b Division of Genetic Therapeutics, Center for Molecular Medicine, Jichi Medical University Shimotsuke Tochigi 329-0498 Japan; c Department of Molecular Oncology, Kyoto University Graduate School of Medicine Kyoto Japan; d Advanced Therapeutic Target Discovery, Department of Gastroenterology, Kobe University Graduate School of Medicine Japan

## Abstract

Identifying cell type-specific molecular markers on the cancer cell surface is essential for understanding cancer progression and for discovering critical neoantigens relevant to immunotherapy. Nucleic acid aptamers serve as powerful tools for probing complex and dynamic cell surface characteristics. Here, we introduce a streamlined comparative aptamer profiling methodology that enables side-by-side analysis of cell surface remodeling. Using cell-SELEX (systematic evolution of ligands by exponential enrichment), we generated a modified base-incorporated aptamer library directly from cells, which was then employed to explore the surface states of normal and mutant protein-expressing cells. Differential analysis of aptamer enrichment using next-generation sequencing revealed distinct aptamer signatures that correlated with cell types. Our analysis demonstrated that mutant K-Ras expression dynamically altered cell surface composition. Individual aptamers showed specific binding to mutant K-Ras-expressing cells without requiring sequence optimization. Moreover, target identification of one aptamer revealed abnormal translocation of a mitochondrial matrix protein to the cell surface without detectable changes in mRNA or protein levels upon altered cellular signaling. These findings highlight the dynamic modulation of cell surface states by aberrant cellular signaling. Overall, we present a useful comparative strategy to investigate cell surface alterations. This approach may help uncover previously unrecognized cell surface markers associated with oncogenic signaling.

## Abstract 2

Comparative aptamer profiling was established to examine cell surface molecular states. This approach revealed bidirectional alterations and unexpected surface localization of a mitochondrial protein under oncogenic signaling.

## Graphical abstract

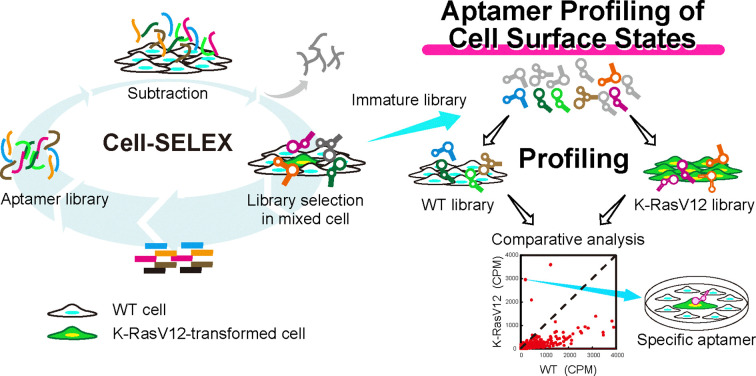

An aptamer is a single-stranded DNA or RNA molecule with a unique sequence and higher-order structure that recognizes a variety of biomolecules with high specificity and strong affinity. Aptamers are developed from synthesized random nucleic acid libraries through SELEX, an *in vitro* molecular evolution technology.^1,2^ SELEX can be employed not only to develop aptamers specific to predefined target molecules but also to generate aptamers targeting undefined molecular complexes, such as cell membranes^3^ and tissue samples.^4^ One promising method to investigate cell surface-targeting aptamers is called cell-SELEX.^5–8^ Although cell-SELEX has been widely used, discovering candidate aptamers that specifically bind to the target cell remains challenging and is usually a laborious and time-consuming process to obtain aptamers of interest. Therefore, an easy and efficient methodology is anticipated to achieve identification of target aptamers of interest.

Cancer biomarkers have emerged as pivotal tools in oncology research, offering insights into the molecular mechanisms underlying cancer biology. Recent advancements in biomarker development have been driven by technological progress; for instance, molecular signatures of various cancer cell types have been explored through differential gene expression profiles^9,10^ and proteomic analyses.^11^ In contrast, fewer challenges have been reported in directly addressing cell surface molecular states in cancer cells with diverse genetic mutations. Understanding the biology of cancer cell surfaces is crucial for comprehending the characteristics and behaviors of cancer cells and will play a pivotal role in the advancement of cancer biomarkers and immunotherapies.^12^ However, analyzing the cell surface molecular signatures remains technically challenging. Therefore, the development of cutting-edge technology and the subsequent generation of knowledge about cancer cell surface biology are anticipated.

In this study, we present a comparative aptamer profiling methodology to obtain cell surface state information and to identify target cell-specific binding aptamers through simple differential profiling analysis. We demonstrate that aptamer profiling analysis of K-RasV12-transformed MDCK cells uncovered significant alterations in cell surface molecular states. Detailed analysis using one of the candidate aptamers found that dysregulated cellular signaling resulted in the emergence of a noncanonical cell surface protein observed in mutant K-Ras- and mutant Src-transformed cells. Our results suggest that aptamer profiling analysis is a useful approach for investigating changes in cell surface states and has potential for developing cancer cell-targeting aptamers.

## Results

### Preparation of an aptamer profiling library by cell-SELEX

**Fig. 1:**
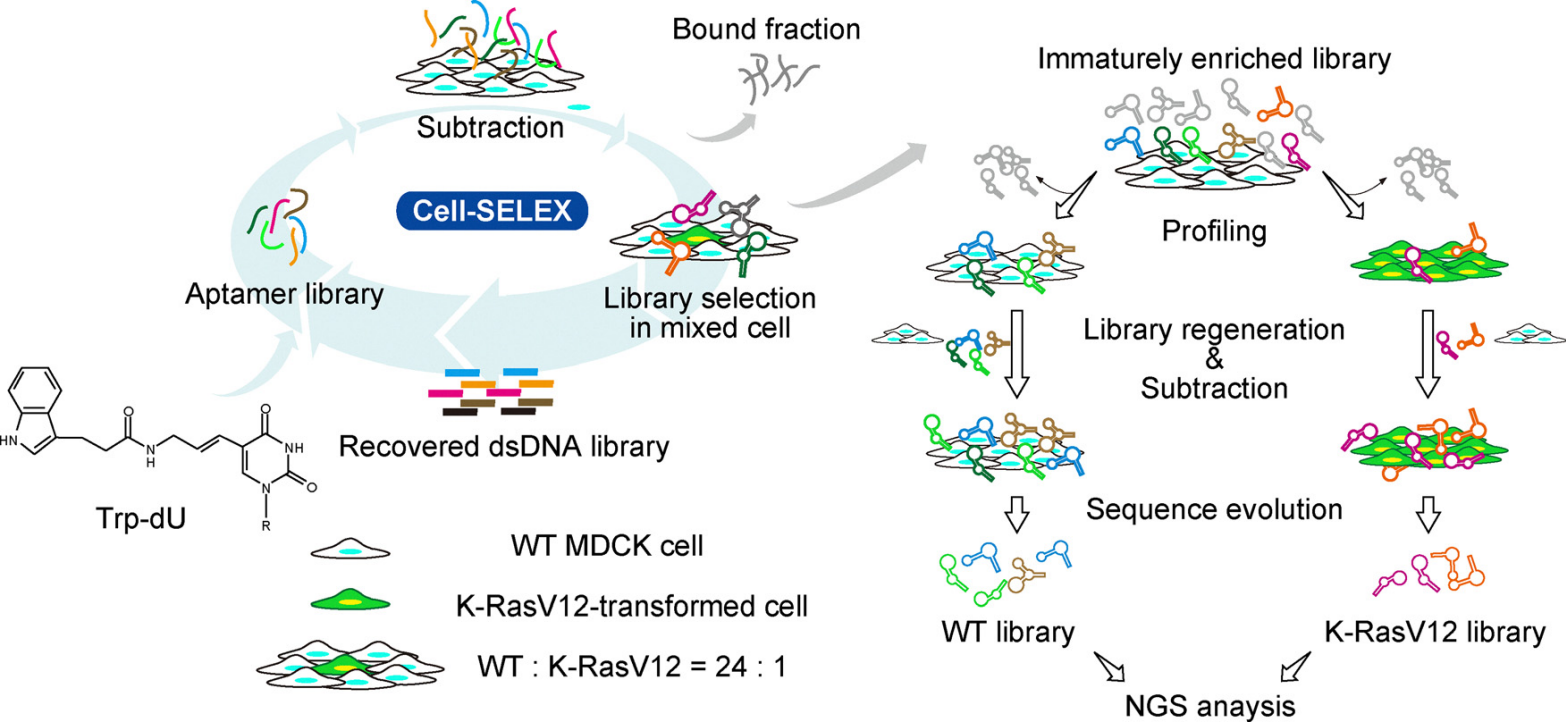
Experimental workflow of aptamer profiling. Crude aptamer library generation was followed by profiling analysis. Trp-dU was used in place of dT to enzymatically generate the modified base-incorporated aptamer library. An immaturely enriched library, obtained after several rounds of cell-SELEX, was used for profiling, followed by the sequence evolution step. The resulting library was analyzed by NGS.

**Fig. 2:**
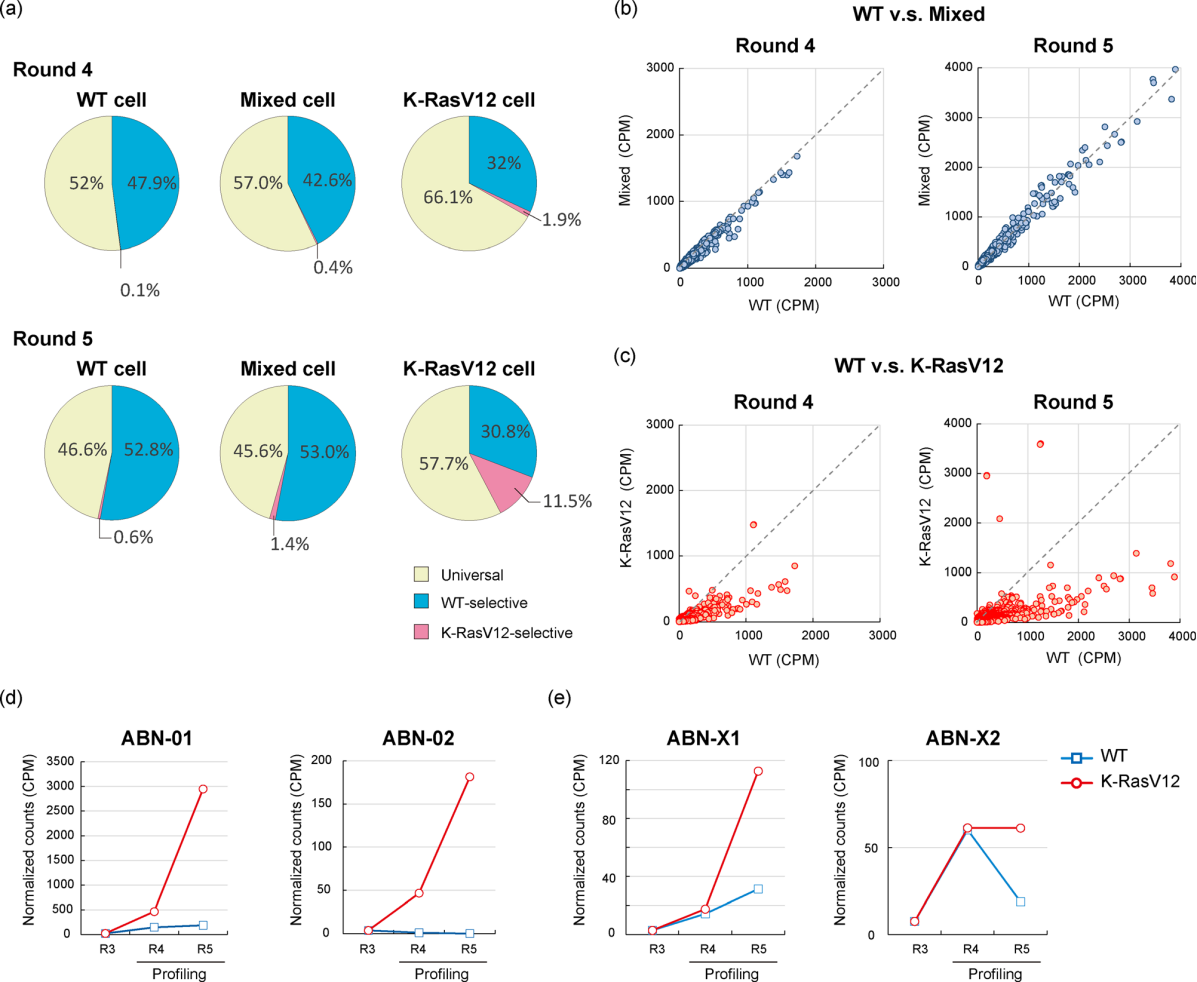
Aptamer profiling of cell surface states. (a) Sequence content analysis of WT, mixed, and K-RasV12 cell libraries after profiling at rounds 4 and 5 is shown. Results of comparative analysis of aptamer profiles between (b) WT and mixed cells and (c) WT and K-RasV12 cells at the same round are shown. Profile changes of representative sequences (d) ABN-01 and ABN-02, or (e) ABN-X1 and ABN-X2, are shown. Similar sequence profiles between WT and mixed cells support the technical reproducibility of the profiling procedures. CPM: counts per million.

The strategy of cell-SELEX for generating an immaturely enriched crude aptamer library, along with subsequent cell surface profiling analysis, is depicted in Fig. 1. Cell-SELEX was performed using empty vector-transformed control MDCK cells (WT), GFP-fused K-RasV12-transformed MDCK cells (K-RasV12), and a mixture of these cells (mixed). The single-stranded DNA (ssDNA) library was enzymatically synthesized to incorporate tryptamino-dU (trp-dU) instead of dT to enhance DNA aptamer functionality by introducing artificial hydrophobic residues (hereafter, aptamers contain trp-dU unless stated otherwise).^13^ Negative selection using WT MDCK cells was performed in all steps before exposing the aptamer library to each cell type. The mixed cell was used during the generation of the aptamer library to approximate an environment of cancer development^14^ and to analyze differences in cell surface states between WT and K-RasV12 cells. Following extensive washing, the ssDNA library was recovered and amplified. The crude aptamer library, obtained after the third round of selection, was employed for cell surface aptamer profiling. In round 4, the ssDNA library was exposed to WT cells, K-RasV12 cells, and mixed cells. Each library was then recovered and amplified independently. For round 5, each ssDNA library from round 4 was exposed again to the same cell type as in round 4 (sequence evolution step). Libraries prepared for next-generation sequencing (NGS) were analyzed using the Illumina MiSeq system (Fig. 2 and Fig. S1). The average output for each sample was about 1 million reads. Raw sequencing data were processed to extract sequences with intact lengths of the random region using forward and reverse primer sets. The normalized frequency of each sequence in the total sample reads was calculated and compared across selection rounds and cell types. For data analysis, we extracted sequences that displayed consistent enrichment across consecutive selection rounds (from round 3 to round 4, and/or from round 4 to round 5), thereby excluding those with minimal or no growth that are more likely to represent random or stochastic amplification events.

### Analysis of cell surface aptamer profiles

Sequence content analysis identified three main types: universal aptamers that bound equally well to both WT and K-RasV12 cells, WT-selective aptamers that exhibited a two-fold stronger binding to WT cells compared to K-RasV12 cells, and K-RasV12-selective aptamers that bound at least twice as strongly to K-RasV12 cells compared to WT cells. The WT library was occupied with universal aptamers (46.6%) and WT-selective aptamers (52.8%) at round 5 (Fig. 2a: WT cells). The mixed cell library exhibited the same sequence contents as the WT library (Fig. 2a: mixed cells). In contrast, although the population of universal aptamers was dominant (57.7%) at round 5 of the K-RasV12 library, the content of K-RasV12-selective aptamers increased from 1.9% at round 4 to 11.5% at round 5 (Fig. 2a: K-RasV12 cells). Because only 0.6% were K-RasV12-selective aptamers in the WT library at round 5, these results indicated that differential sequence evolution quickly occurred from the same starting library.

Analysis of cell surface aptamer profiles for WT, K-RasV12, and mixed cells was conducted. Comparisons between WT cells and mixed cells revealed almost identical aptamer profiles at rounds 4 and 5 (Fig. 2b). Given that about 96% of the cells in the mixed cell system were WT cells, this result suggested that the presence of a small percentage of K-RasV12 cells did not alter the overall cell surface properties of the surrounding WT cells at an easily detectable level.

In contrast, the aptamer profile of K-RasV12 cells differed significantly from that of WT cells (Fig. 2c). The frequencies of many major sequences decreased in the K-RasV12 library, and this trend became more pronounced by round 5 of the selection process. Since mutant K-Ras expression was strongly induced after doxycycline addition, insufficient mutant K-Ras expression was ruled out as a cause for the reduced library reactivity (Fig. S2). Moreover, because no new sequences emerged that would break the balance of sequence abundance in the library, the decreased frequency could be attributed to reduced interactions between aptamers and targets on the K-RasV12 cell surface compared with WT cell surface. Some possible causes that affect aptamer binding activity can be considered, such as changes in cell surface protein levels and glycosylation patterns,^15^ but the exact mechanism remains to be elucidated.

Furthermore, the nearly identical sequence profiles between the WT and mixed libraries demonstrate that the selection procedure is highly reproducible and not biased by stochastic amplification, thereby validating the comparative profiling approach (Fig. 2b). In contrast, the WT and K-RasV12 libraries showed distinct sequence evolution patterns despite starting from the same library (Fig. 2c). These findings indicate that library evolution is determined by the cellular context rather than by the experimental procedure, ensuring that the observed differences reflect comparative profiling of the cell surface.

Sequence content analysis showed that K-RasV12-selective aptamers first appeared at round 4 and rapidly increased in content by round 5 (Fig. 2a). As the profiling rounds progressed, the differences in frequencies between the WT and K-RasV12 libraries became more pronounced (Fig. 2c), indicating that these aptamers suggested preferential binding to K-RasV12 cells compared with WT cells. This led to selective sequence enrichment and increase in their relative frequencies in the aptamer library as the cell population shifted from a mixed population, with only 4% K-RasV12 cells, to exclusively K-RasV12 cells. Additionally, some aptamers that initially showed no significant difference or low read counts at round 4 exhibited a rapid increase by round 5 in the K-RasV12 context, highlighting the significance of sequence evolution in uncovering minor candidates embedded within the library (Fig. 2d and e). Given that the starting library was derived from a mixed cell population containing only 4% K-RasV12 cells, these findings suggest that aptamer profiling can identify even minor aptamers that evolve under complex conditions.

### Validation study of aptamer profiling results

**Fig. 3:**
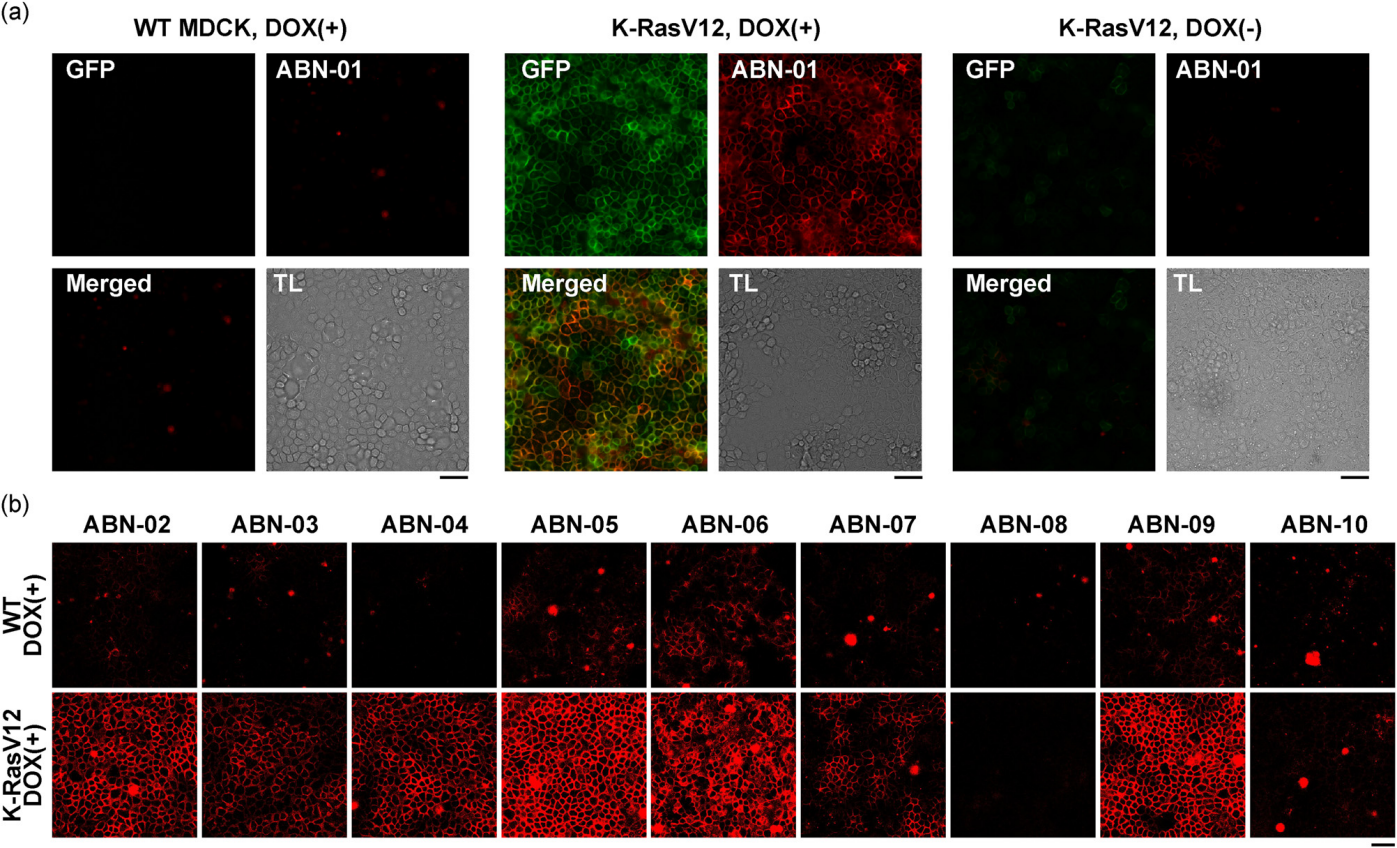
Analysis of aptamer staining in WT and K-RasV12 cells. The cells were incubated with 100 nM TYE665-labeled aptamers for 30 min at 25 °C before analysis. (a) Representative images of aptamer staining with the TYE665-labeled ABN-01 aptamer are shown. (b) Screening results for the top candidate aptamers are shown. ABN-08 and ABN-10 showed weak binding to K-RasV12 cells as indicated by the quantitative results in Fig. S3. Experiments were performed independently at least twice (*n* = 2 for most aptamers, and quantitative data are shown as supporting information due to limited replicates). In the aptamer staining images, GFP-fused K-RasV12 is shown in green, TYE665-labeled aptamer in red, and transmitted light (TL) in gray. Scale bar: 50 μm.

To validate the results of the aptamer profiling analysis, the top 10 candidates among the K-RasV12-selective aptamers that were rapidly growing in the K-RasV12 library were selected (Table 1, ABN series). We selected K-RasV12-selective aptamers for the validation study because an increasing marker is generally preferred over decreasing one as a molecular marker. These aptamers had unique sequences and showed frequencies at least 5 times higher in the K-RasV12 library (Table 1). Fluorescent dye-conjugated aptamers were enzymatically synthesized and used to label cells, followed by microscopic analysis (Fig. 3 and Fig. S3). Imaging analysis was performed under the same conditions as the selection process, without fixation or nuclear staining, because the aptamers were not cross-linked by PFA and the DNA staining dye could interfere with binding of DNA aptamers. Imaging analysis revealed that ABN-01 recognized only K-RasV12 cells and not WT cells (Fig. 3a). The distinct staining pattern of ABN-01, compared to the fluorescence of GFP-fused K-RasV12, indicated that the aptamer tightly bound to the cell surface rather than resulting from GFP fluorescent leakage or being diffusely distributed throughout the cell. Because the scramble control showed no binding to any of the cells tested (Fig. S4), the observed binding can be attributed to ABN-01 itself. To eliminate the possibility that the focal plane for WT cells was incorrect due to the absence of positional reference data in WT cells, Hoechst nuclear staining was performed prior to aptamer binding (Fig. S5). ABN-01, but not the scramble control, exhibited clear binding to the cell surface of DOX-induced K-RasV12, confirming that the microscopic focus was correctly set on the cells for the analysis (Fig. S5). Moreover, ABN-01 binding specificity was investigated using the mixed cell condition, demonstrating highly specific binding to K-RasV12 cells (Fig. S6).

**Table 1:** Candidate aptamer sequences. Normalized counts at each round and fold change values for K-RasV12 cells are shown. Sequences correspond to the random domain only. The ranking score was defined based on the sequence frequency in Round 5 of the K-RasV12 cell library. The lowercase “t” indicates the position of the modified nucleotide Trp-dU. CPM: counts per million

Sequence ID	Random domain	Rank at R5	R3	Normalized counts (CPM)				Fold change (K-RasV12/WT)	
				WT		K-RasV12			
				R4	R5	R4	R5	R4	R5
ABN_01	GCGGCtCGtGGGtCtACGtGGCCGAGAGGACGAGGGGGCtGCC	6	24	148	189	467	2949	3.16	15.58
ABN_02	GAAGGAGCGAGGACGGGCtGGGACGtCGtGGGtCtACGCCGGA	146	4	1	0	47	181	45.74	>100
ABN_03	CGGCCACtACGCGAGAGGGGGGAtCGtGGGtCtACGAGAAtCC	420	4	5	5	32	86	6.29	16.53
ABN_04	GAAAGGtGAGCGGACAGGAGtCGtGGGtCtACGACGAGACCGA	491	2	2	2	32	75	15.72	36.28
ABN_05	GACCGAtCGtGGttCtACGAGCGGCGGGACCCGAGGAGtAAGC	660	7	8	2	28	61	3.45	29.39
ABN_06	CCGGGGGGCGGGtACCACtCGtGGGtCtACGAGttGCtGGAGG	735	6	9	3	17	56	1.80	18.06
ABN_07	GGACGCGtCGtGGAtCtACGACGGGGAGGGGCGGAGtCAtCCt	793	4	8	6	24	53	2.98	8.57
ABN_08	CCGGCGCGGACAAACAGCtCAGAGAAGGGGCAtCAGGCGCACC	1199	1	1	7	5	39	4.76	5.38
ABN_09	CCGGtCGCCGGCCAGCGAGGCCCtCtCGtGGGtCtACGAGACA	1482	6	8	1	20	33	2.50	32.15
ABN_10	GCCAAGCCCAGGACtGCGtCGGCAAACCCCCGCGCGCGGCGtG	1338	1	0	4	2	36	>100	8.73

Additionally, ABN-01 did not bind to cells without doxycycline (DOX) induction, underscoring the role of abnormal signaling triggered by K-RasV12 expression in the aptamer's recognition of K-RasV12 cells (Fig. 3a and Fig. S4 and S5). To assess whether ABN-01 binding is dependent on K-RasV12 signaling, we employed different MDCK cell systems in which mutant Src was transformed.^16^ Aptamer staining analysis showed that ABN-01 also recognized mutant Src-expressing MDCK cells (Fig. S7). This result suggests that ABN-01 recognized changes in cell surface states modulated by aberrant cellular signaling driven by K-RasV12 expression, rather than directly recognizing unique changes specific to K-RasV12 mutation.

The accuracy of the aptamer profiling was verified. Eight out of the ten selected ABN aptamers showed higher specificity to K-RasV12 cells over WT cells, indicating the high accuracy of the profiling results for developing unique aptamers that bind specifically to target cell types of interest (Fig. 3b and Fig. S3). To confirm the results of the aptamer binding assay, we also performed flow cytometry to investigate ABN aptamer interactions (Fig. S8). Although most surface-bound aptamers were removed after trypsin treatment, residual signals were still detected by flow cytometry and subsequently analyzed. This analysis revealed stronger binding and/or internalization to K-RasV12 for most ABN aptamers, but not for scramble control, ABN-08 or ABN-10, in agreement with the results of the aptamer binding assay (Fig. 3 and Fig. S8). Given that most selected ABN aptamers were of low abundance in both WT and K-RasV12 libraries at rounds 4 and 5 (Table 1), it can be inferred that the candidate aptamer selection process would have been technically difficult and time-consuming without comparative profiling analysis as demonstrated here. Taken together, the aptamer profiling methodology is an efficient approach for broadly investigating cell surface binding aptamers.

### Target molecule identification of ABN-01

**Fig. 4:**
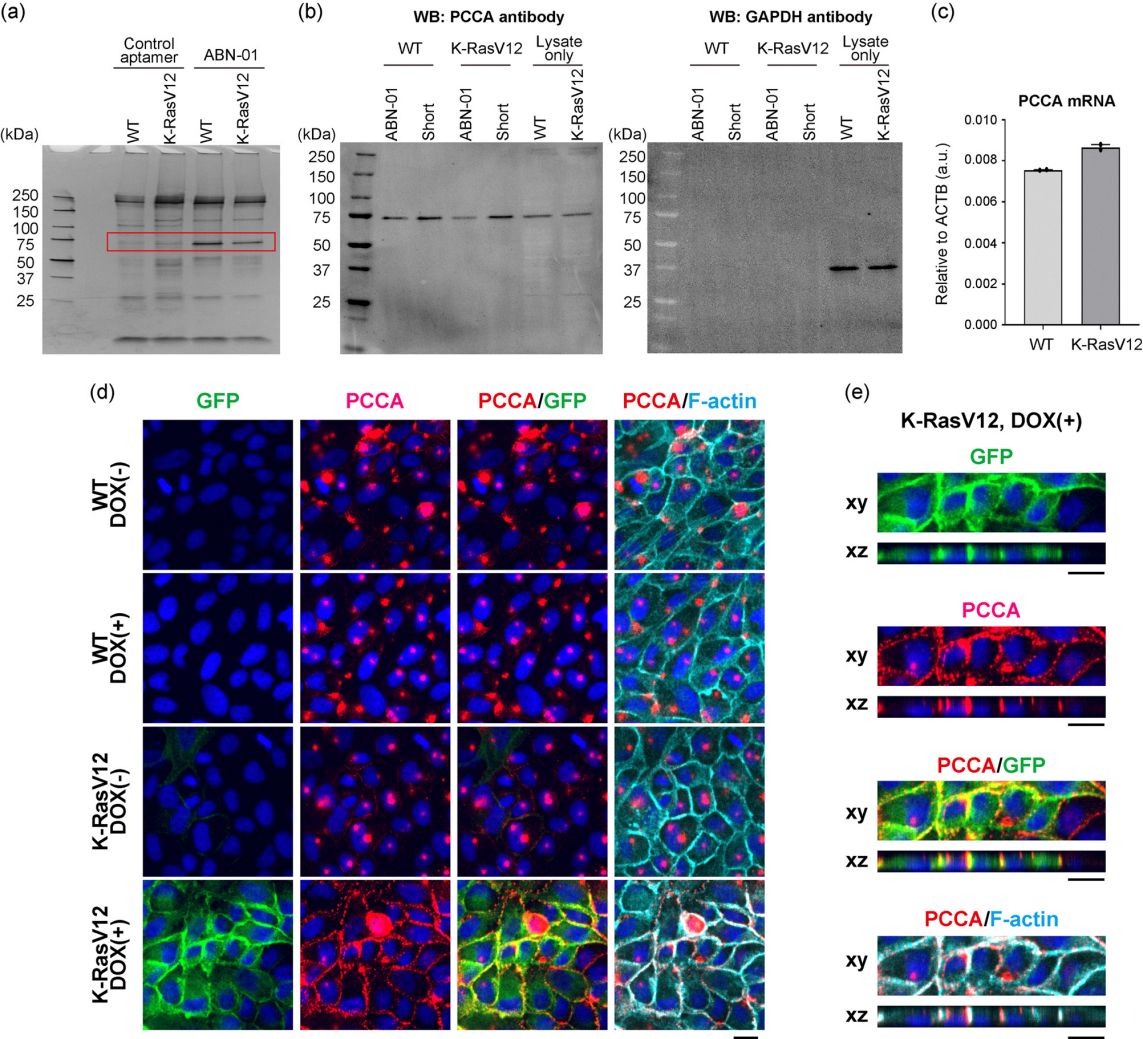
Target identification of the ABN-01 aptamer. (a) Silver-stained SDS-PAGE image from aptamer-based precipitation analysis of cell lysates. The red rectangle indicates the region analyzed by mass spectrometry. Shown is a representative result. (b) Fluorescence western blot analysis using a monoclonal anti-PCCA antibody to validate the mass spectrometry results. An ABN-01 aptamer and a reverse primer domain-removed ABN-01 aptamer (lane: short) were used for target precipitation. Precipitated samples and crude cell lysates (lane: lysate only) were analyzed, and the same gel was also analyzed for GAPDH as a control. A fluorescently labeled secondary antibody was used for band detection. Shown is a representative result. (c) Quantitative PCR analysis of PCCA mRNA, with data normalized to ACTB mRNA levels. (d) Immunofluorescence analysis of PCCA localization on the cell surface using a polyclonal anti-PCCA antibody under non-permeabilized conditions. Representative images are shown. In these images, GFP-fused K-RasV12 is shown in green, PCCA in red, F-actin (SiR-Actin) in cyan, and DNA in blue. (e) A detailed view of PCCA localization at the cell surface in K-RasV12 cells under non-permeabilized conditions, as shown in the K-RasV12-DOX(+) panel of (d). Scale bar: 15 μm. All experiments were independently repeated three times with similar results, except for panel c (qPCR, *n* = 2).

The target molecule for the ABN-01 aptamer was identified to gain a deeper understanding of the biology underlying the cell surface remodeling of K-RasV12-transformed cells. Enzymatically synthesized biotinylated ABN-01 and the chemically synthesized control aptamer (Table S1) were used to precipitate a target protein from cell lysates using streptavidin-coated beads (Fig. 4a). SDS-PAGE analysis of precipitates showed a major band specifically observed for the ABN-01 aptamer. Analysis of the band using LC-MS yielded a single reliable candidate protein, propionyl-CoA carboxylase subunit α (PCCA) (Fig. S9). Western blot analysis of ABN-01 aptamer-based precipitates by using the anti-PCCA antibody confirmed the MS results (Fig. 4b).

Interestingly, while PCCA is a mitochondrial enzyme that catalyzes the carboxylation of propionyl-CoA to methylmalonyl-CoA and typically localizes in the mitochondria, aptamer-based staining of K-RasV12-transformed cells indicated its localization at the plasma membrane (Fig. 3). Given that induced aberrant intracellular signaling in K-RasV12 cells, upregulation of PCCA protein expression was expected; however, western blot analysis of cell lysates showed no change in PCCA protein levels between WT and K-RasV12 cells (Fig. 4b, lane: lysate only). Consistently, qPCR analysis revealed no change in PCCA mRNA levels (Fig. 4c). Because ABN-01 bound equally well to PCCA protein from both WT and K-RasV12 cells (Fig. 4a and b), it is unlikely that post-translational modifications affected ABN-01 binding efficiency. Therefore, these data suggested altered PCCA translocation to the cell surface in response to K-RasV12 expression. This hypothesis was further evaluated by immunostaining analysis using an anti-PCCA antibody on non-permeabilized cells, which minimizes intracellular staining and facilitates visualization of plasma membrane localization. Immunostaining revealed that PCCA colocalized with GFP-K-RasV12 and F-actin (SiR-Actin staining) at the cell surface in K-RasV12 cells but was not observed in WT cells (Fig. 4d and e and Fig. S10). The localization was absent or very weak without DOX induction, suggesting that K-RasV12 expression influenced the PCCA localization pattern. Immunostaining of permeabilized cells clearly revealed the expression and localization of PCCA in the cytoplasm of both K-RasV12 and WT cells (Fig. S11), indicating that abnormal K-Ras signaling leads to a noncanonical localization of PCCA at the cell surface without changes in the levels of PCCA mRNA or protein.

### Sequence analysis of ABN-01 and other candidate aptamers

**Fig. 5:**
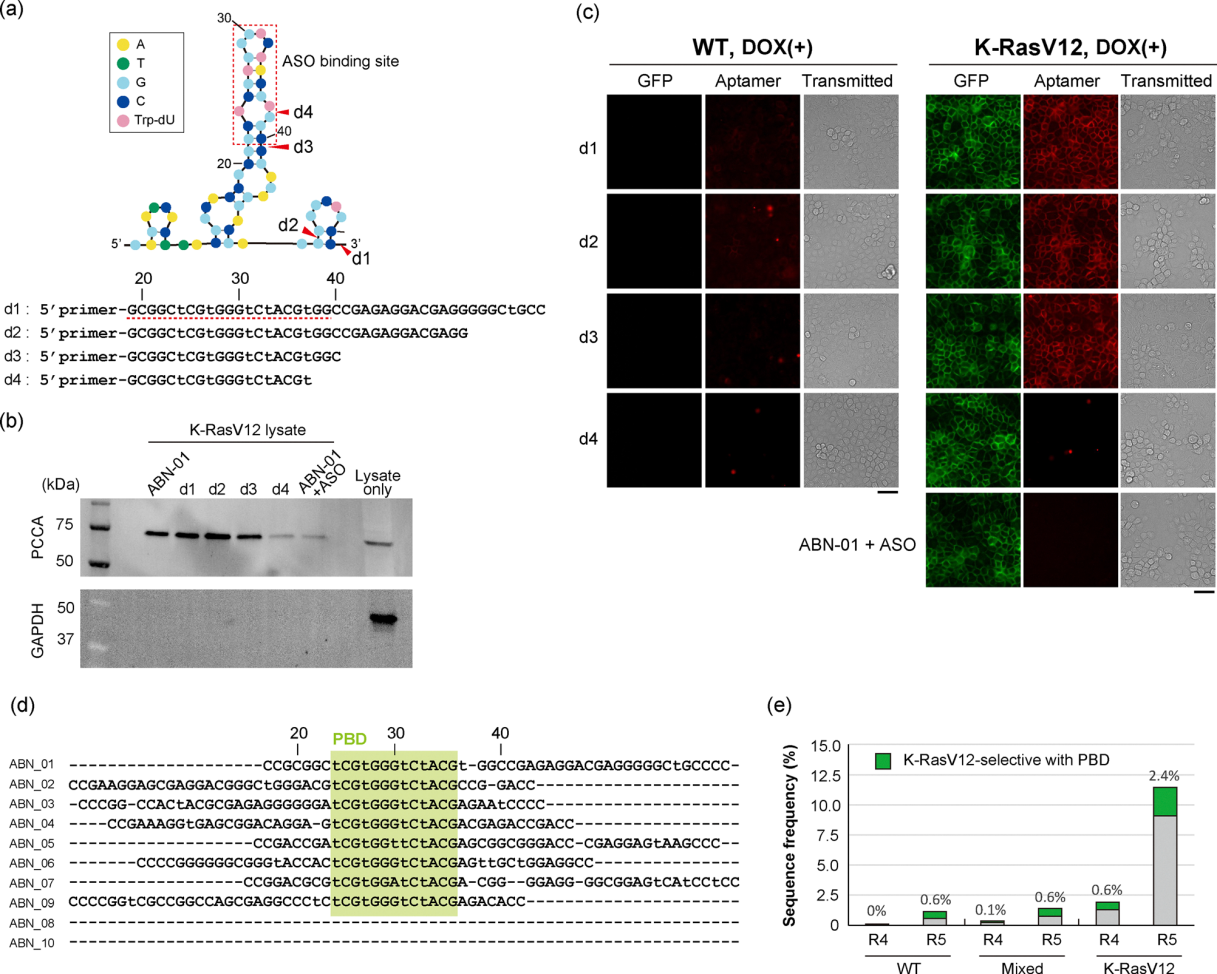
Domain analysis of the ABN-01 aptamer. (a) Predicted secondary structure diagram of the ABN-01 aptamer. Truncated sequences and truncation positions are indicated as d1, d2, d3, and d4. The ASO binding domain is indicated by a red dashed rectangle and line. Lowercase “*t*” in the sequence indicates the position of the modified nucleotide Trp-dU. (b) The binding activity of ABN-01 variants to the target PCCA protein was analyzed by aptamer-based precipitation followed by fluorescence western blot analysis. K-RasV12 lysate in the right lane was used as a control (lane: lysate only). A representative result from two independent experiments is shown. (c) Aptamer staining analysis of WT and K-RasV12 cells using TYE665-labeled ABN-01 variants, including the ASO-inactivated ABN-01 aptamer. The cells were incubated with 100 nM TYE665-labeled aptamers for 30 min at 25 °C prior to analysis. Representative images from three independent experiments are shown. In the images, GFP-fused K-RasV12 is shown in green, TYE665-labeled aptamer in red, and transmitted light in gray. Scale bar: 50 μm. (d) Result of sequence homology analysis of the top 10 candidates is shown with the PBD region highlighted in green. (e) Frequency changes along selection rounds are shown for the K-RasV12-selective aptamer. The frequency of the PBD-containing sequence is shown in green, with percentage values displayed above the bars.

To understand the ABN-01 aptamer structure and functional domain, various forms of the ABN-01 aptamer were synthesized (Fig. 5a). Aptamer precipitation and aptamer staining analyses of WT and K-RasV12 cells demonstrated that binding patterns of ABN-01 variants to PCCA correlated with the variants’ ability to label K-RasV12 cells (Fig. 5b and c), supporting direct interaction of ABN-01 with PCCA at the cell surface. Moreover, the core binding domain responsible for the interaction with PCCA (PCCA binding domain; PBD) was investigated based on the binding assays of ABN-01 variants. The binding of ABN-01 to PCCA was inhibited by an antisense oligonucleotide (ASO) complementary to this domain (Fig. 5a–c). Additionally, a mutation in the loop around the ASO-binding region reduced the binding ability of the aptamer to PCCA (Fig. S12). These observations indicated the importance of the aptamer structure and PBD sequence for its binding ability to PCCA. These results provided further support of the interaction of ABN-01 with PCCA on the cell surface.

The sequence homology of the top 10 candidates was analyzed based on the identified binding region of ABN-01. Alignment analysis of the candidates identified PBD as a 13-nucleotide sequence in the stem-loop region that was highly conserved throughout the candidate sequences (Fig. 5d), suggesting that most top candidate ABN aptamers would target the same molecule as ABN-01. Sequence analysis of the libraries obtained after aptamer profiling showed that PBD-containing sequences were quickly enriched only in the K-RasV12 library (Fig. 5e). Although further detailed analysis of other candidates is still necessary, *in silico* sequence analysis supported the hypothesis that PCCA serves as a unique molecular marker in K-RasV12 expressing MDCK cells.

## Discussion

In this study, we introduce a streamlined comparative methodology, aptamer profiling analysis, enabling side-by-side analysis of dynamic changes in cell surface molecular states. This approach enables the discovery of alterations in cellular characteristics, offering detailed information of cell surface remodeling. Aptamer profiling analysis of K-RasV12-transformed MDCK cells uncovered dynamic alterations in both increased and decreased cell surface molecular signatures in response to mutant K-Ras expression. Identified aptamers exhibited sufficient binding specificity without requiring any sequence optimization, underscoring the streamlined characteristics of the platform. Subsequent detailed analysis using the K-RasV12 MDCK cell-targeting aptamer ABN-01 revealed unexpected ectopic translocation of mitochondrial protein PCCA to the plasma membrane without quantitative changes in PCCA mRNA and protein. These results suggest that aptamer profiling analysis of cell surface states has the potential to reveal previously unrecognized cell surface remodeling associated with aberrant cellular signaling.

Noncanonical cell surface localization of intracellular proteins is considered a naturally occurring phenomenon during cancer development. For instance, intracellular proteins such as nucleolin,^17^ ribosomal protein,^18^ and plectin^19^ have been identified as translocated to the cancer cell surface, making them attractive targets for anti-cancer drug development. Notably, mitochondrial ATP synthase has been reported to localize on the plasma membrane of certain cells (referred to as ectopic ATP (eATP) synthase), where it plays a key role in extracellular ATP accumulation.^20–22^ The translocation of eATP synthase to the cancer cell surface occurs *via* DRP1 and KIF5B along microtubules.^23^ Since mutant RAS causes DRP1-mediated mitochondrial fragmentation,^24^ K-RasV12 in our cell system may facilitate the noncanonical localization of PCCA by inducing aberrant mitochondrial fission and transport to the cell surface. Furthermore, analysis of plasma membrane fractions revealed that mitochondrial matrix proteins were also detected on the cancer cell surface,^23^ further supporting our observations of the noncanonical translocation of mitochondrial matrix protein PCCA. These findings underscore the need for a more detailed analysis of endogenous systems to clarify whether mutant Ras expression contributes to PCCA mislocalization. In this context, PCCA may serve as a valuable case study to investigate noncanonical translocation mechanisms, as only a limited number of such proteins have been reported. Elucidating the molecular mechanisms governing cancer cell surface remodeling and aberrant localization of intracellular proteins will be crucial to deepen our understanding of the underlying cancer biology.

Although we focused on few aptamers in this study, additional aptamer candidates without the PBD sequence were identified in the K-RasV12 library. These different aptamers may recognize distinct targets other than that of the ABN01 aptamer. It would be of interest to explore this point using these aptamers to expand our knowledge regarding cell surface remodeling. Additionally, employing different types of modified bases in the aptamer library may broaden the range of detectable targets, as varying chemical properties and structural components can confer different binding characteristics to the aptamers. While we only used tryptamine modification in this study to introduce hydrophobic properties, several other types of modified bases could potentially be utilized for the aptamer profiling as long as the aptamer library can be enzymatically constructed. Therefore, our approach may serve as a flexible and broadly applicable tool for exploring various aspects of the cell surface with the potential to contribute to the discovery of biomarkers and therapeutic targets.

In profiling analysis, we observed reduction of profiling scores of many aptamers in the K-RasV12 library, suggesting that the molecular profile of the plasma membrane changed dynamically in both increasing and decreasing directions in response to aberrant cellular signaling. It is unknown whether cell surface remodeling to reduce molecular states of K-RasV12 cells observed in this study is directly associated with cancer biology, or if they are merely consequences of aberrant signaling. However, it has been reported that autophagy-dependent degradation of MHC-I causes a reduction in cell surface MHC-I levels, leading to immune evasion in pancreatic cancers.^25^ Furthermore, aberrant localization of membrane protein Scribble to the cytoplasm has been associated with resistance to KRAS inhibitors.^26^ Inhibition of the molecular mechanisms behind these molecules has been shown to reverse drug-resistant cancer cells into sensitive ones. These findings highlight the importance of not only the increase but also the decrease of factors on the cancer cell surface. An in-depth analysis of the molecular biology underlying cell surface remodeling will be essential for a better understanding of cancer development, potentially leading to the creation of innovative therapeutic strategies and novel cell surface biomarkers.

## Conclusion

In conclusion, we demonstrated that aptamer-based efficient and comparative profiling analysis is a useful methodology not only for developing aptamers targeting specific cell types of interest but also for deciphering cell surface molecular biology with genetic mutations of interest. Because investigation of cell surface remodeling remains technically challenging, this approach will contribute to our understanding of cellular behavior and intercellular communication associated with altered cellular signals. Moreover, aptamer profiling may provide a potential examination platform to discriminate cell conditions based on diverse cell surface signatures. We propose that aptamer profiling analysis represents a promising approach to facilitate the development of cancer cell-targeting drug delivery platforms, neoantigens for cancer immunotherapy, and diagnostics based on dynamic alterations of cancer cell surface states due to genetic mutations.

## Author contributions

J. K. and K. E. performed the biochemical and microscopic experiments. K. Ohba performed preliminary experiments for cell-SELEX and obtained initial microscopic data. J. K. and K. Ohba equally contributed to the work. H. O. performed RNA-seq experiments. K. Okahara. and H. S. supported microscopic data acquisitions. M. S., Y.F., and T. I. supervised the project. Y. S. conceived, designed, and supervised the project, analyzed the data, and wrote the manuscript. All authors contributed to the manuscript.

## Conflicts of interest

The research described in this study was conducted at KAN Research Institute, Inc., with funding provided by Eisai Co., Ltd. The authors J. K., H. O., K. Okahara, K. E., and Y. S. are currently employees of Eisai Co., Ltd. These authors and H. S., K. Ohba, and M. S. declare no conflicts of interest related to this work. The authors T. I. and Y. F. received grant support from Eisai Co., Ltd. and served as consultants for the company. However, they had no role in the analysis or interpretation of the study results.

## Supplementary Material

CB-OLF-D5CB00110B-s001

## Data Availability

The data supporting this article have been included as part of the supplementary information (SI). Supplementary information: Additional figures showing aptamer profiling data, validation experiments, and specificity analyses, as well as tables listing oligonucleotides and SELEX conditions. See DOI: https://doi.org/10.1039/d5cb00110b.
