# Supplementary material for: Comparative aptamer profiling reveals cell surface remodeling and the emergence of a noncanonical cell surface protein under oncogenic signaling
Source: RSC Chem Biol. 2025 Oct 3;6(12):1941–9. doi: 10.1039/d5cb00110b (PMC12541808; doi:10.1039/d5cb00110b)
Supplement: CB-006-D5CB00110B-s001 [file CB-006-D5CB00110B-s001.pdf]

Supplementary Information (SI) for RSC Chemical Biology.
This journal is © The Royal Society of Chemistry 2025

Supplementary Information for

Comparative Aptamer Profiling Reveals Cell Surface Remodeling and the Emergence of a Noncanonical Cell Surface Protein Under Oncogenic Signaling

Jungo Kakuta, ‡ § [a] Kenji Ohba, ‡ [b] Hideaki Ogasawara, § [a] Kyohei Okahara, § [a] Kazumi Emoto, § [a] Hiroaki Sako, [c] Miho Sekai, [c] Yasuyuki Fujita, [c] Toshio Imai [a, d] and Yogo Sakakibara § *[a]

Contents:

Materials and Methods

Figure S1. Scatter plot of aptamer profiling data.

Figure S2. Western blot analysis of GFP-K-RasV12 expression.

Figure S3. Staining analysis of candidate aptamers.

Figure S4. Aptamer staining of WT and K-RasV12 cells with and without DOX induction.

Figure S5. Aptamer staining with Hoechst nuclear staining.

Figure S6. Specificity analysis of ABN-01 and ABN-02 aptamers in mixed cell conditions.

Figure S7. Aptamer staining of WT and mutant Src-transformed MDCK cells.

Figure S8. Flow cytometry analysis of aptamer interaction.

Figure S9. List of proteins identified by mass spectrometry.

Figure S10. Immunofluorescent analysis for cell surface localization of PCCA.

Figure S11. Immunofluorescent analysis for localization of PCCA in permeabilized sample.

Figure S12. Domain analysis of the ABN-01 aptamer.

Table S1. Oligonucleotides used in this study.

Table S2. Cell-SELEX conditions used in this study.

Materials and Methods

Materials

Reagents	Manufacture	Identifier
**Cell culture**		
D-PBS(-)(1x)	FUJIFILM Wako Pure Chemical	045-29795
Collagen I Cellware 96 Well Black/Clear Plate, With lid	CORNING	356649
0.25% Trypsin-EDTA(1x)	gibco	2520005
Cellmatrix I-P	Nitta Gelatin	638-00661
HCl	wako	084-05425
Doxycycline Hyclate	Nacalai tesque	19088-71
**Template medium**		
D-MEM (Hight Glucose) with Phenol Red	FUJIFILM Wako Pure Chemical	045-30285
Fetal Bovine Serum (LOT# 14L368)	Nichirei Biosciences	172012-500ML
GlutaMAX™-1(100x)	gibco	35050-061
Penicillin-streptomycin(x100)	FUJIFILM Wako Pure Chemical	168-23191
**Components for WT MDCK culture medium**		
Template medium		
Blasticidin	InvivoGen	ant-bl
**Components for KRas, cSrc culture medium**		
Template medium		
Blasticidin	InvivoGen	ant-bl
Zeocin	InvivoGen	ant-zn
**Antibodies**		
PCCA polyclonal Antibody[Rabbit / IgG]	Invitrogen	PA5-100169
PCCA monoclonal Antibody[Mouse / IgG1]	Invitrogen	MA5-27386
Anti-Pan-Ras(Ab-3)mouse mAb (Ras 10)	Calbiochem	OP40-100UG
AffiniPure Rabbit Anti-Mouse IgG (H+L)	Sigma-Aldrich	315-005-045
Goat Anti-Rabbit IgG StarBright blue 700	BIO RAD	1200416
Goat Anti-Mouse IgG StarBright Blue 700	BIO RAD	12004159
Anti-GAPDH hFAB Rhodamine Antibody	BIO RAD	AbD22549
Alexa Fluor® 647 AffiniPure $F(ab')_2$ Fragment Donkey Anti-Rabbit IgG (H+L)	Jackson ImmunoResearch	711-606-152
**Reagents**		
10xD-PBS(-)	FUJIFILM Wako Pure Chemical	048-29805
5-Indolyl-AA-2'-deoxyuridine-5'-Triphosphate	TriLink	N-2065-0250
dATP, dCTP, dGTP, dTTP	Promega	U1330
Streptavidin Agarose Ultra Performance	Vector Laboratories	N1000-002
Sodium Dextran Sulfate	Nacalai	10930-36

NP-40 (IGEPAL CA630)	SIGMA	I3021
8mol/L Sodium Hydroxide Solution	FUJIFILM Wako Pure Chemical	194-09575
6mol/L Hydrochloric Acid	FUJIFILM Wako Pure Chemical	084-05425
Magnesium chloride Hexahydrate	FUJIFILM Wako Pure Chemical	135-00165
Chloroform	FUJIFILM Wako Pure Chemical	038-02606
Ethanol (99.5)	FUJIFILM Wako Pure Chemical	057-00456
30% Acrylamide/Bis Solution, 29:1	BIO RAD	1610156
Ethidium bromide	Invitrogen	15585-011
MiSeq Reagent Kit v3 (150 Cycle)	illumina	MS-102-3001
TRIZOL	Invitrogen	15596018
Mineral oil	Sigma Aldrich	M8410-1L
Span 80	Sigma Aldrich	S6760-250ML
Tween 80	Sigma Aldrich	P8074-100ML
Triton-X 100	Wako	168-11805
KOD+Neo enzyme	Toyobo	KOD-401
NucleoSpin Gel and PCR Clean-up	Macherey-Nagel	740609.25
FG Streptavidin Magnetic Beads	TAMAGAWA SEIKI	TAS8848N1170
Halt Protease Inhibitor Single-Use Cocktail(100x), 24 x 100µl microtubes	Thermo scientific	1860932
4x Laemmli Sample Buffer	BIO RAD	1610747
Precision Plus Protein All Blue Standards	BIO RAD	1610373
Methanol	FUJIFILM Wako Pure Chemical	137-01823
Silver Stain MS Kit	FUJIFILM	299-58901
1 mol/L(±)Dithiothreitol solution	FUJIFILM Wako Pure Chemical	044-33871
Difco skim Milk	BD	23100
10% Tween 20	BIO RAD	1610781
10x Tris/Glycine/SDS Buffer	BIO RAD	1610772
Trans-Blot Turbo Mini-size LF PVDF Membrane	BIO RAD	1704274
Trans-Blot Turbo Mini-size Transfer Stacks	BIO RAD	1704274
Mini-PROTEAN TGX Stain-Free Gels	BIO RAD	4568096
Trans-Blot Turbo 5x Transfer Buffer	BIO RAD	1704274
4% Paraformaldehyde phophate buffer solution	Nacalai tesque	09154-14
Bovin Serum Albumin Fraction V	SIGMA-Aldrich	10735086001
Hoechst 33258 solution	Dojindo	343-07961
RNeasy Mini Kit	QIAGEN	74104
PrimeScript RT reagent Kit	Takara Bio	RR036A
PowerUp SYBR Green Master Mix	Thermo Fisher	A25742

Cell culture

The original MDCK cell was established by Dr. Walter Birchmeier. MDCK cells that were used in this report were provided by Dr. Yasuyuki Fujita. MDCK cells stably contained empty pTR vector (MDCK-pTR) or stably expressing GFP-K-RasV12 (MDCK-pTR GFP-K-RasV12) and GFP-cSrcY527F (MDCK-pTR GFP-cSrcY527F) in a tetracycline-inducible manner[14, 16, 27]. Cells were cultured as described in previous report[14]. WT cells were cultured in template medium (DMEM high glucose with 10% FBS, 1% penicillin-streptomycin, 1% GlutaMAX) with 0.5 µg/mL blasticidin at 37 ºC in a 5% CO_2 moist environment. K-RasV12 cells were cultured in template medium with 0.5 µg/mL blasticidin and 40 µg/mL zeocin at 37 ºC in a 5% CO_2 moist environment.

Cell-SELEX

A single-stranded DNA (ssDNA) library with a random region of 43 nucleotides including Trp-dU instead of dT was enzymatically synthesized as following. Nucleotide sequence used in this study is listed in Table S1. 5'-Biotinylated library template was mixed with forward primer at 1:1.5 ratio. Primer extension solution was prepared using KOD-Plus-Neo DNA polymerase with separately prepared dNTP mix which included Trp-dUTP instead of dTTP. The solution was mixed with DNAs and the volume was adjusted to 50 µL. Primer extension reaction was performed using thermal cycler (95 ºC/3 min, 55 ºC/10 sec, 68 ºC/30 min). Streptavidin agarose beads in 200 µL of PBSN (PBS with 0.005% NP-40) was added to the reaction mixture and incubated at 16 ºC for 15 min with gentle shaking. After PBSN washing, 40 µL of 20 mM NaOH solution was added and incubated at 37 ºC for 1 min with vigorous shaking. Dissociated ssDNA library was collected and the dissociation step was performed again. Total 80 µL of ssDNA solution was neutralized by 14 µL of 80 mM HCl solution. The ssDNA was precipitated by ethanol precipitation and dissolved in deionized water.

For cell-SELEX, the synthesized ssDNA library was denatured at 95 °C for 3 min then slowly cooled down to room temperature over 30 min to form stable ssDNA structures in buffer PBSM (PBS with 1 mM magnesium chloride). The renatured ssDNA library solution was diluted to an

appropriate volume with PBSM. The library solution was mixed with competitors in advance to mixing with cells if needed. For screening, 6×10^5 cells or 3×10^5 cells were plated onto collagen-coated 12-well or 24-well plates, respectively (Corning), followed by doxycycline induction (final 2 μg/mL) for 16 hours before use (Table S2). Cells for both subtraction and screening were treated with blocking competitor oligos prior to adding ssDNA library. The medium for WT MDCK cells was exchanged for the library mixture and incubated for subtraction at room temperature for 10 min. The supernatant was then incubated with mixed cells. Screening conditions are in Supporting table 1. After the incubation, cells were washed with PBSM and lysed by 400 μL (12-well) or 200 μL (24-well) of Trizol reagent. The ssDNA was recovered according to the manufacturer's instructions.

The recovered ssDNA was subjected to PCR amplification. Note that droplet PCR was employed for all PCR steps to suppress biased sequence amplification. Hundred μL of PCR solution including the recovered ssDNA was mixed with 250 μL of oil (4.5% Span80, 0.4% Tween 80, 0.05% Triton-X 100, and 95.05% Mineral Oil). The solution was vigorously mixed by magnetic stirrer until completely mixed. PCR was performed first with small volume to determine PCR cycle, then the ssDNA was amplified using the determined cycles. PCR product was analyzed by 6% PAGE electrophoresis followed by ethidium bromide staining. The reaction was mixed with 2.5-time volume of chloroform to extract water layer. The extracted solution was purified by NucleoSpin Gel and PCR Clean-up.

The amplified DNA was diluted to the concentration of 1 ng/μL to prepare template DNA sample for aptamer library generation by primer extension. Two microliters of the solution were used for 100 μL of each PCR amplification. In this PCR amplification step, 5'-biotinylated antisense strand primer was employed, and the number of PCR cycle was fixed to 8 cycles. The biotin-labeled dsDNA product was subjected to primer extension by using 5'-TYE665 fluorophore-labeled primer and dNTP mix including Trp-dUTP instead of dTTP. The ssDNA library purification step was the same as described above. The aptamer library was dissolved

in 10 μL of deionized water and quantified by NanoDrop. The integrity of the regenerated library was analyzed by 8M urea denaturing PAGE. The aptamer library was used for the next cycle of selection.

The PCR products obtained at each round were PCR amplified in a 2-step process to prepare for next generation sequencing analysis by MiSeq (illumina). Adapter sequence primer set was used in the first step followed by using index sequence primer set in the second step. Thermal cycling was performed according to the manufacturer's instruction. The PCR products were purified by NucleoSpin Gel and PCR Clean-up and quantified by Bioanalyzer (Agilent). The products were analyzed by MiSeq according to manufacturer's instructions. All sequencing data for each round were generated as FASTAQ files. After extracting aptamer domain by trimming 5'- and 3'-primer regions, all sequences were used for frequency analysis to investigate generation of aptamers during selections.

Aptamer staining

For aptamer staining analysis, 0.6×10^5 WT cells or 0.25×10^5 K-RasV12 cells were plated onto collagen-coated 96-well plate (Corning). After doxycycline induction for 16 hours, culture medium was replaced with PBSM and blocked with competitor DNAs for 10 min at room temperature. After removing blocking solution, 100 nM TYE665-labeled cloned aptamer in PBSM was added to a well. After 30 min incubation at room temperature, cells were washed with PBSM 2 times, then added 100 μL of PBSM. Images were acquired soon after washing by using BZ-X710 microscope (Keyence) or Dragonfly 200 confocal microscope (Andor Technology). Data were acquired from 2 to 3 different points in each well. The fluorescence images reported here are representative of three independent experiments. Data were visualized by ImageJ. The binding of aptamers to the cell surface was quantified as follows. Line intensity profiles were extracted in Fiji (ImageJ) along straight lines drawn perpendicular to the plasma membrane. For z-stacks, the focal plane with the highest mean intensity in the aptamer channel was identified. For WT cells, the plane was determined based on the transmitted light

image. Profiles from that plane and two adjacent planes above and below were averaged. Background was estimated as the mean of the distal 10% at both ends of each profile and subtracted. Peak height was defined as the background-corrected maximum intensity.

Aptamer precipitation analysis

For preparing cell lysate, 6.0×10^5 cells were plated onto collagen-coated 100 mm dish (Corning). Cells were cultured until confluency. The medium was exchanged with a new medium containing 2 μg/mL doxycycline to induce GFP-K-RasV12 expression over 16 hours. Lysis buffer (1% NP-40 and protease inhibitor cocktail in PBS) was added, and the cells were scraped using a cell scraper. Cell lysates were subjected to mechanical shearing by passing them through a 20-gauge syringe on ice. After centrifugation at 15,000 rpm for 3 minutes at 4 ºC, the supernatant was collected and further centrifuged at 15,000 rpm for 5 minutes at 4 ºC. The supernatant was collected and stored at -80 ºC. Hundred μL of cell lysate was mixed with 50 nM of renatured 5'-biotinylated aptamer, 0.75% dextran sulfate, and 4 μM competitor in 200 μL PBSMN buffer. After 10 min incubation at 25 ºC, 2 μL of pre-washed FG Streptavidin Magnetic Beads were added. The mixture was incubated for 20 min at 16 ºC with gentle shaking. Beads were separated by magnetic stand (Tamagawa Seiki) and thoroughly washed twice with PBSMN containing 0.75% dextran sulfate. Precipitated samples were dissolved in 2x Laemmli buffer and subjected to SDS-PAGE. Protein bands were visualized using silver stain MS kit according to manufacturer's instruction. Excised bands were analyzed by Japan Proteomics using nano-LC/MS/MS and the provided processed peptide identification results were used in this study without further modification. Additional in-house bioinformatic processing, such as analysis of raw spectra, FDR estimation, or replicate analyses, was not conducted. The candidate protein identified by MS was subsequently validated by independent aptamer-based precipitation and Western blotting. For Western blotting analysis following aptamer-based precipitation, proteins were transferred onto a polyvinylidene difluoride (PVDF) membrane using the Transfer Blot Turbo 2 system (Bio-Rad). The membrane was washed twice with PBS-T (PBS

containing 0.02% Tween-20) for 5 minutes each, and then blocked with 5% skim milk in PBS-T for 1 hour at room temperature. After two quick rinses in PBS-T, the membrane was washed three times: first for 15 minutes, followed by two 5-minute washes. The membrane was then incubated with monoclonal anti-PCCA antibody, diluted 1:2000 in 5% skim milk in PBS-T, for 1 hour with gentle shaking. Following two quick rinses and three 5-minute washes in PBS-T, the membrane was incubated with the secondary antibody, diluted 1:2500 in 5% skim milk in PBS-T, for 20 minutes with gentle shaking. After three additional 5-minute washes in PBS-T, protein bands were detected using the ChemiDoc Touch MP imaging system (Bio-Rad) with fluorescent detection.

Immunostaining analysis

Cells were cultured as in aptamer staining analysis. After doxycycline induction for 16 hours, cells were fixed with fresh 4% paraformaldehyde in PBS. Fixed cells were washed 3 times with PBS, followed by blocking with BSA-PBST (1% BSA and 0.1% Tween-20) for 10 min at room temperature. Polyclonal anti-PCCA antibody, diluted 1:400 or 1:1,200 in BSA-PBST, was incubated with cells for 1 hour at room temperature or 3 hours on ice, respectively. Cells were washed 3 times with PBS and then incubated with secondary antibody, diluted 1:400 in BSA-PBST, and 5 ug/mL Hoechst 33258 for 1 hour at room temperature in the dark. To visualize the cell surface, cells were incubated with 100 nM SiR-Actin (CY-SC001, Spirochrome), which labels F-actin, for 1 h at room temperature. After staining, cells were washed three times with PBS to remove excess probe. Images were acquired using Dragonfly 200 confocal microscope (Andor Technology). Data were visualized by ImageJ.

Quantitative Real-Time PCR

Cells were cultured as described for cell lysate preparation. Total RNA was isolated using the RNeasy Mini Kit. cDNA synthesis was performed using the PrimeScript RT reagent Kit with specific primers for ACTB and PCCA (Table S1). Quantitative PCR (qPCR) was conducted

using PowerUp SYBR Green Master Mix on a QuantStudio System (Applied Biosystems). All procedures followed the manufacturer's protocols.

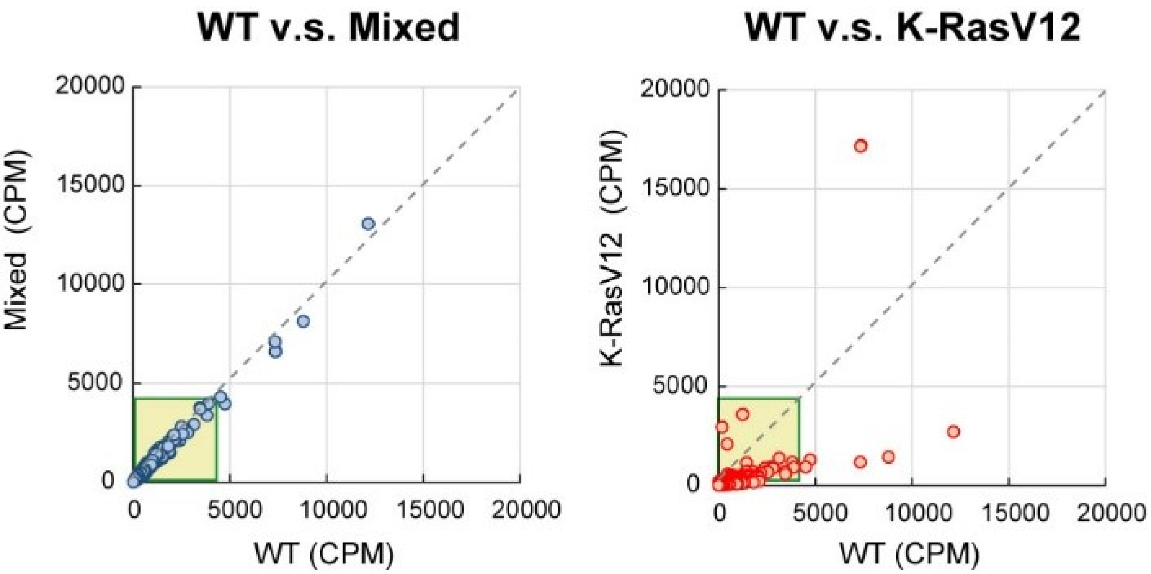

Figure S1. Scatter plot of aptamer profiling data.
Entire version of aptamer profiling data is shown. Green square is the region shown in Figure 2.

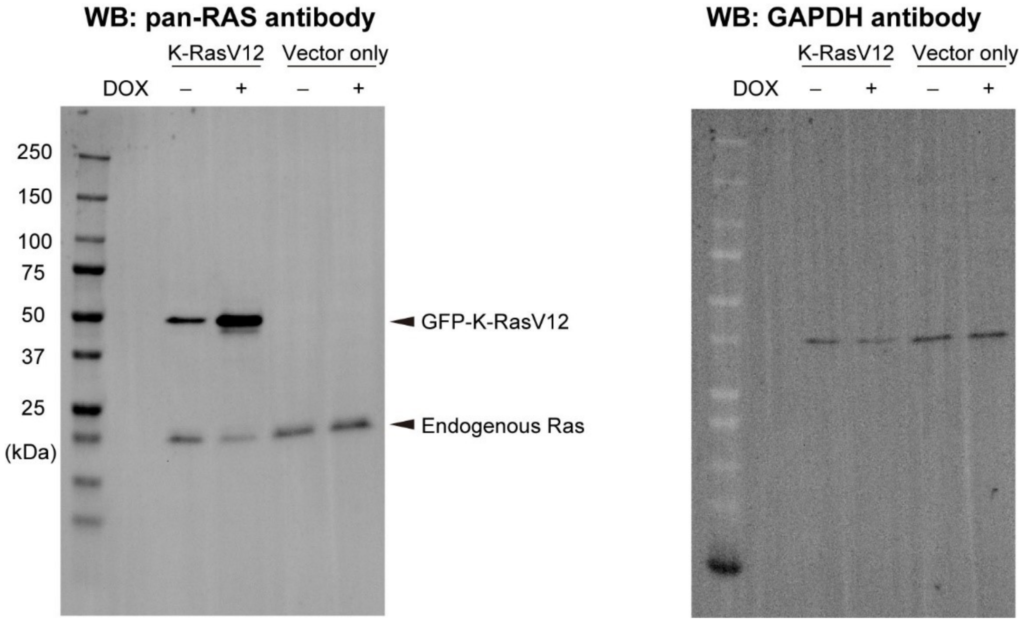

Figure S2. Western blot analysis of GFP-K-RasV12 expression.
Cell lysates prepared from WT and K-RasV12 cells with or without DOX induction were analyzed to validate the level of endogenous Ras and exogenous GFP-fused K-RasV12 expression. Shown is a representative result from two independent experiments.

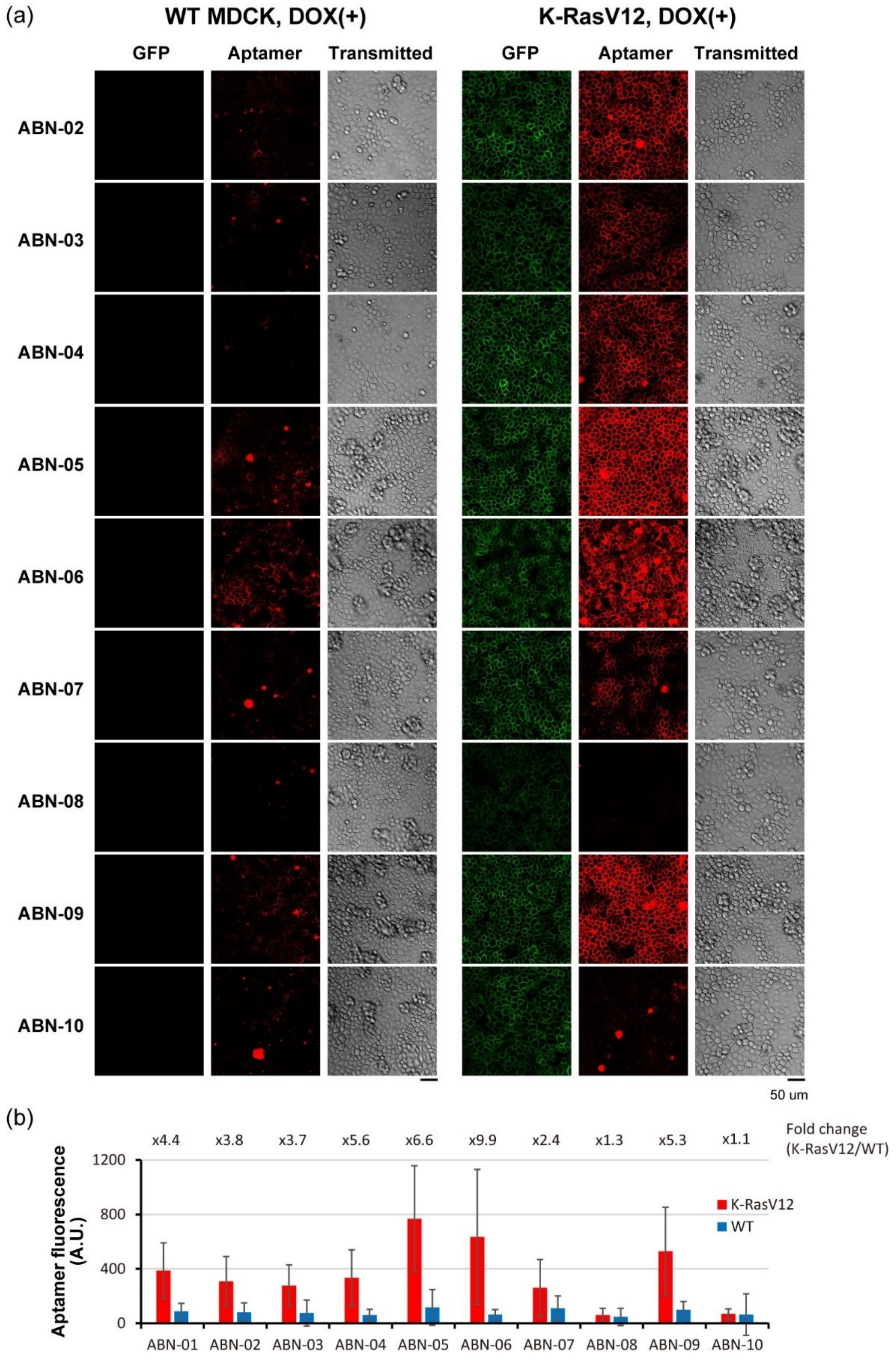
(a)
WT MDCK, DOX(+)
K-RasV12, DOX(+)
GFP
Aptamer
Transmitted
ABN-02
ABN-03
ABN-04
ABN-05
ABN-06
ABN-07
ABN-08
ABN-09
ABN-10
50 um
(b)
Fold change (K-RasV12/WT)
x4.4 x3.8 x3.7 x5.6 x6.6 x9.9 x2.4 x1.3 x5.3 x1.1
Aptamer fluorescence (A.U.)
1200
800
400
0
K-RasV12
WT
ABN-01 ABN-02 ABN-03 ABN-04 ABN-05 ABN-06 ABN-07 ABN-08 ABN-09 ABN-10

Figure S3. Staining analysis of candidate aptamers.
(a) Microscopic analysis of the binding ability of TYE665-labeled candidate aptamer to WT and K-RasV12 cells. Cells were incubated with 100 nM of aptamers for 30 min at 25 ºC prior to analysis. Representative images are shown. In the images, GFP-fused K-RasV12 is shown in green, TYE665-labeled aptamer in red, and transmitted light in gray (b) Quantitative analysis of aptamer binding to the cell surface. Aptamer signals on cell surfaces were measured as line intensity profiles using Fiji (imageJ). The fold change in signal intensity between WT and K-RasV12 cells for each aptamer is shown above the corresponding bar in the graph. Cell numbers analyzed were WT: n=80, 164, 120, 184, 202, 202, 202, 160, 264, 266; and K-RasV12: n=80, 80, 160, 160, 206, 168, 174, 164, 234, 320 (from two independent experiments). Error bars represent standard deviation. For some aptamers (ABN-04 to ABN-10), analyses were based on duplicate experiments (n = 2), and therefore no statistical analysis was performed.

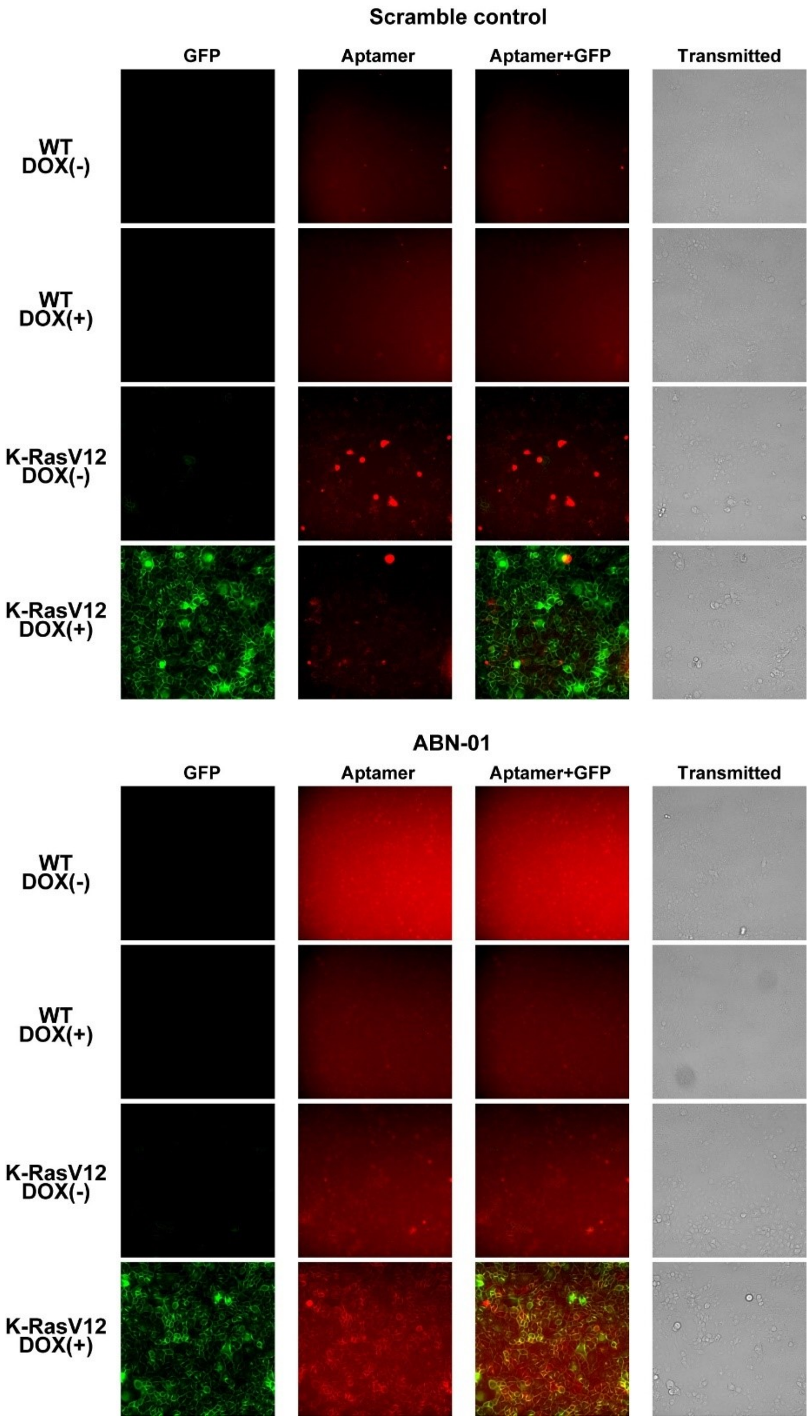

Figure S4. Aptamer staining of WT and K-RasV12 cells with and without DOX induction.

Cells were incubated with 100 nM of aptamers for 30 min at 25 ºC prior to imaging. Representative images from two independent experiments are shown. In the

images, GFP-fused K-RasV12 is shown in green, TYE665-labeled aptamer in red, and transmitted light in gray.

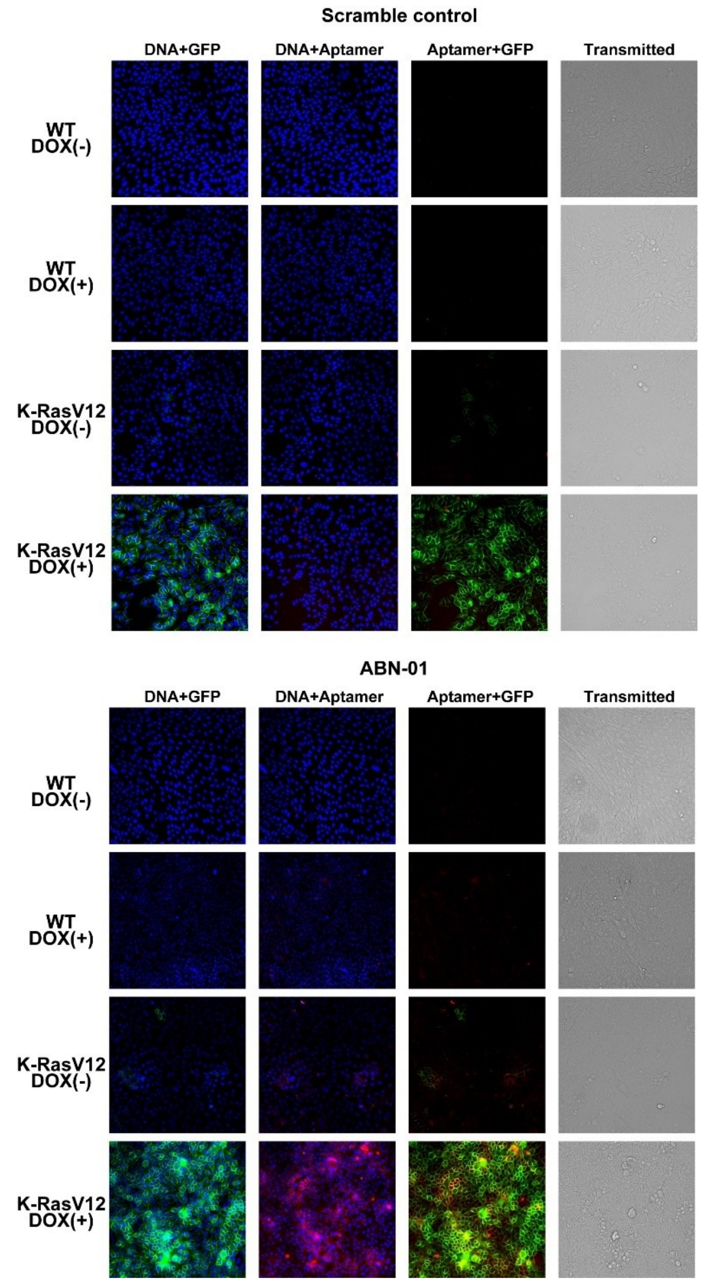

Figure S5. Aptamer staining with Hoechst nuclear staining.
Hoechst staining was performed to provide a positional reference for WT cells lacking morphological markers in aptamer binding analysis. Cells were treated with Hoechst at 37 ºC for 30 min prior to aptamer binding. After Hoechst staining, cells were washed with PBS and incubated with 100 nM of aptamers for 30 min at 25 ºC prior to imaging. Representative images from two independent experiments are shown. In the images, GFP-fused K-RasV12 is shown in green, TYE665-labeled aptamer in red, DNA in blue, and transmitted light in gray.

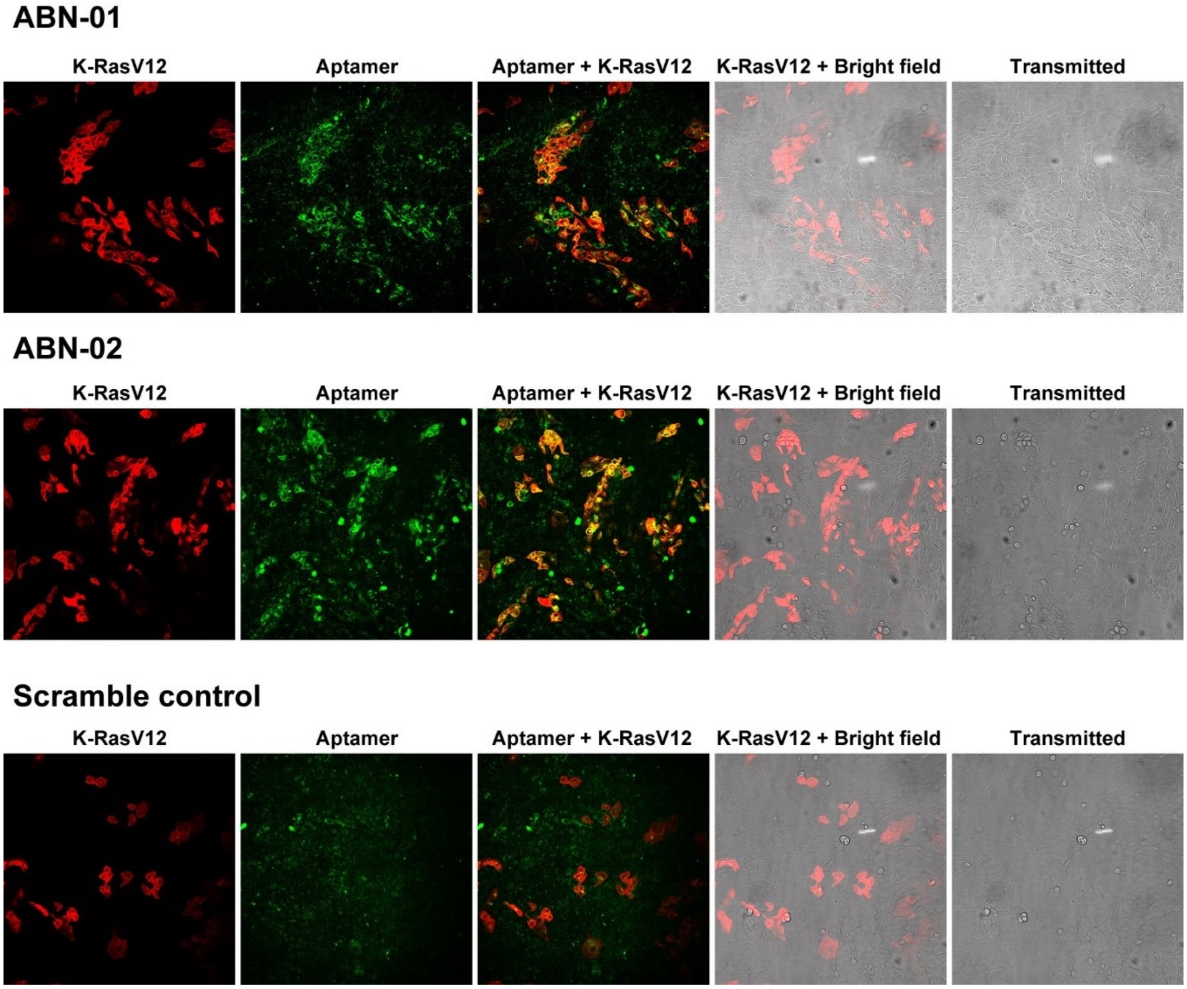

Figure S6. Specificity analysis of ABN-01 and ABN-02 aptamers in mixed cell conditions.

K-RasV12 cells were mixed with WT cells at a 1:10 ratio and incubated with 100 nM of aptamers for 30 min at 25 ºC prior to imaging. Representative images from three independent experiments are shown. In the images, GFP-fused K-RasV12 is shown in red, TYE665-labeled aptamer in green, and transmitted light in gray.

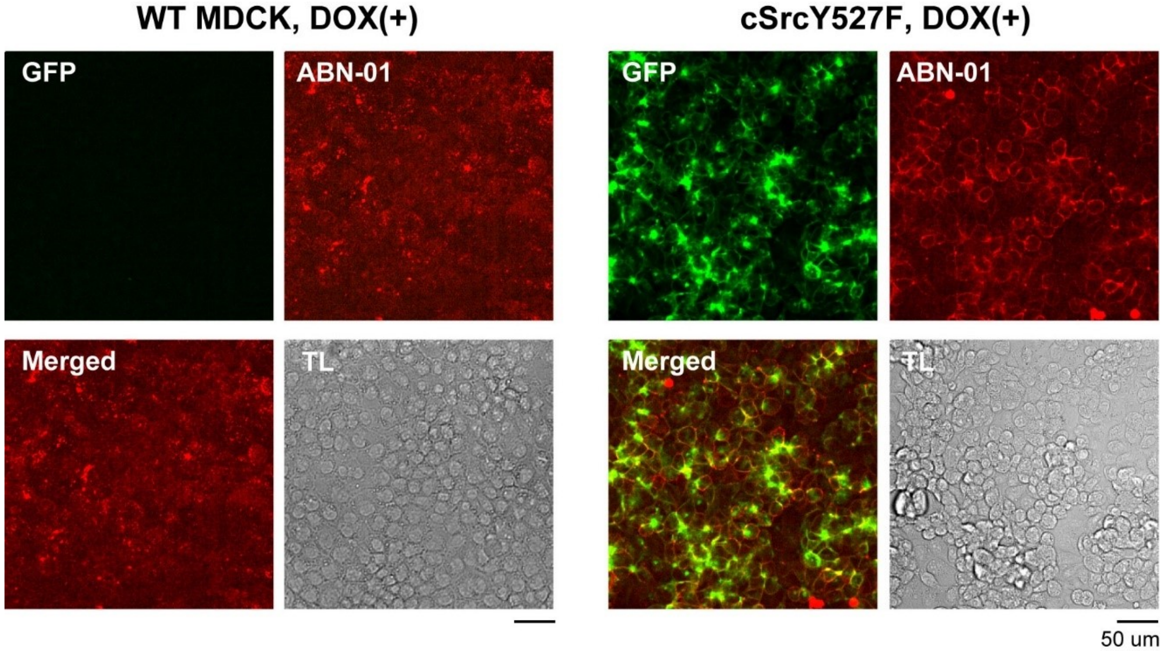

Figure S7. Aptamer staining of WT and mutant Src-transformed MDCK cells. Cells were incubated with 100 nM TYE665-labeled aptamer on ice for 3 hours in culture medium. Since changes in cell shape was observed for Src-transformed MDCK cells at 37 ºC under PBSM, the staining analysis was conducted under on ice condition. Representative images from two independent experiments are shown. In the images, GFP-fused cSrc is shown in green, TYE665-labeled aptamer in red, and transmitted light in gray.

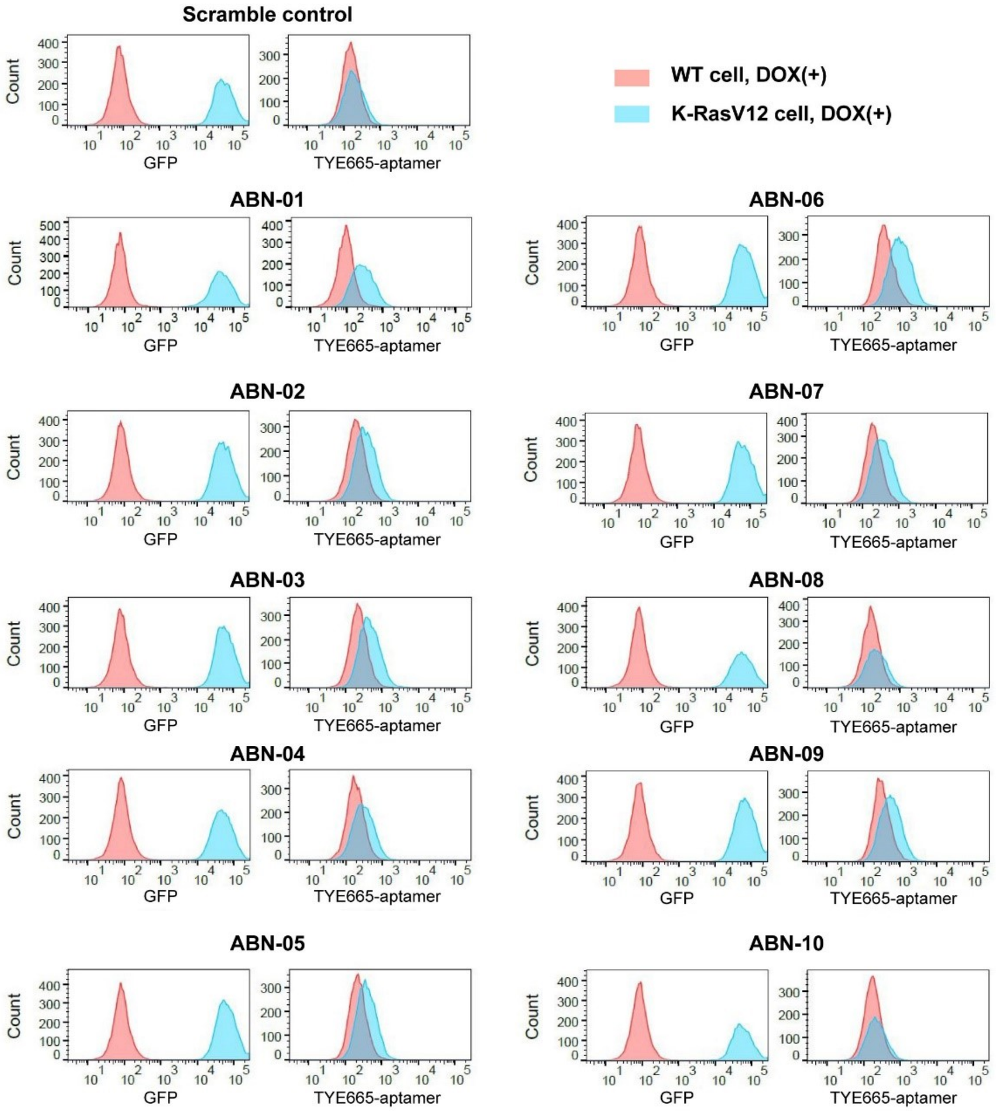

Figure S8. Flow cytometry analysis of aptamer interaction.
Cells were incubated with 100 nM TYE665-labeled aptamer for 20 min at 37 °C. The reaction medium was replaced with PBSM to remove unbound aptamers, followed by a 1 h incubation in PBSM at 37 °C to allow potential internalization of bound aptamers. After extensive washing and trypsin treatment, cells were fixed with 4% PFA and analyzed by flow cytometry. Experiments were performed at least twice independently.

List of proteins identified by MS analysis

Sample Name		Protein	MW	Score	Queries matched	emPAI*
ABN-1	1	propionyl-CoA carboxylase alpha chain, mitochondrial isoform X2 [Canis lupus familiaris]	75,599	290	15	0.71
ABN-1	2	Keratin, type II cytoskeletal 1	63,751	214	7	0.41
ABN-1	3	Keratin, type I cytoskeletal 10	57,676	100	6	0.28
ABN-1	4	keratin, type II cytoskeletal 8 [Canis lupus familiaris]	54,684	83	1	0.07
ABN-1	5	keratin, type II cytoskeletal 3 [Canis lupus familiaris]	62,477	65	1	0.06
ABN-1	6	Keratin, type II cytoskeletal 2 epidermal	64,527	54	2	0.06
ABN-1	7	keratin, type II cytoskeletal 6A isoform X6 [Canis lupus familiaris]	62,832	54	2	0.12
ABN-1	8	Serine protease 1	26,153	39	2	0.15

emAPI : exponentially modified Protein Abundance Index*

Figure S9. List of proteins identified by mass spectrometry (MS) analysis. The list shows the provided processed identification data without further modification. No additional in-house analyses of raw spectra, FDR values, or replicates were performed.

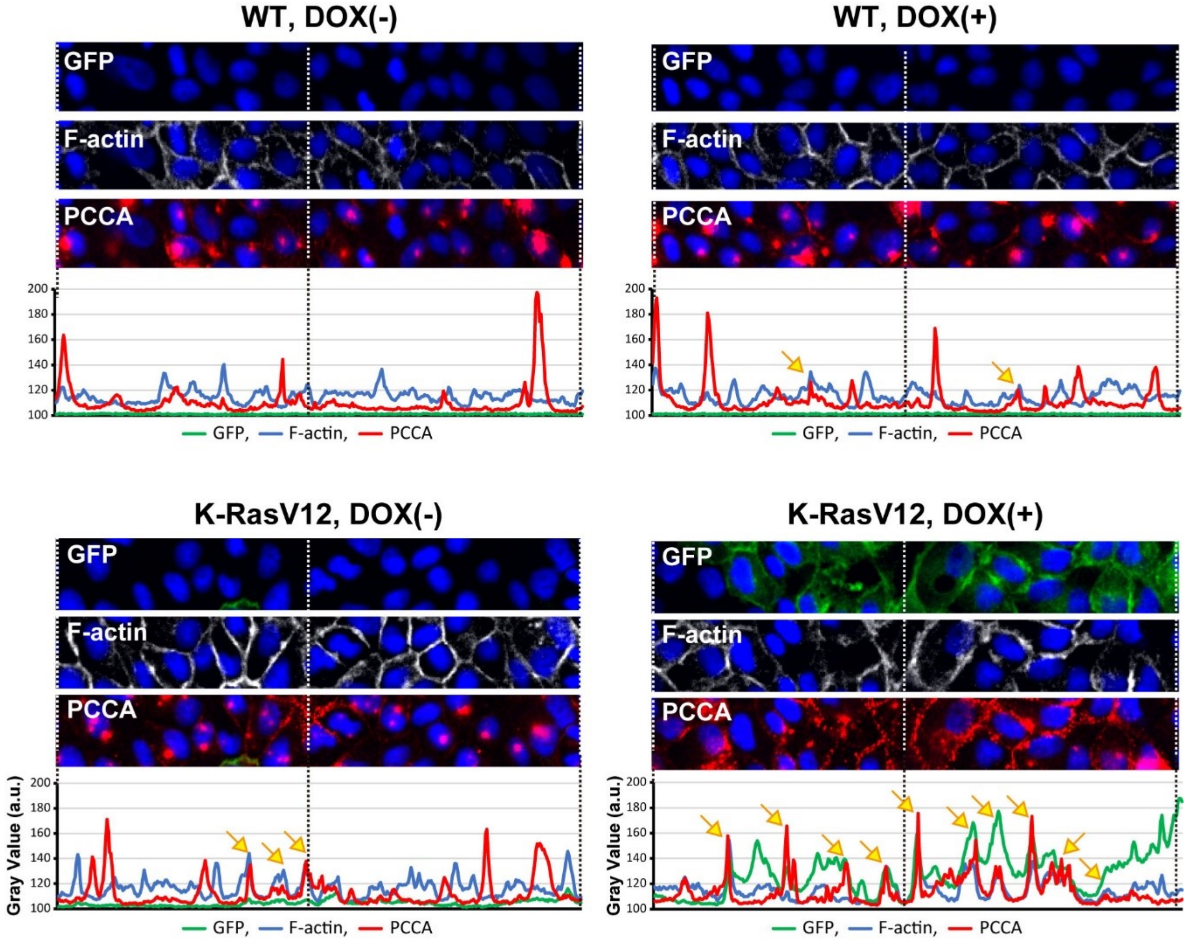

Figure S10. Immunofluorescent analysis for cell surface localization of PCCA. Colocalization of PCCA with F-actin or GFP-K-RasV12 on the plasma membrane was analyzed using ImageJ. Representative images showing the strong localization of PCCA (red), GFP-fused K-RasV12 (green), and F-actin stained by SiR-Actin (gray) at the cell periphery of only the DOX-induced K-RasV12 cells but not WT cells or DOX-negative condition. Colocalization was analyzed using the plot profile function in ImageJ. Overlapping peaks of PCCA and F-actin or GFP-K-RasV12, indicated by yellow arrows, show colocalization at the membrane. Representative images from two independent experiments are shown.

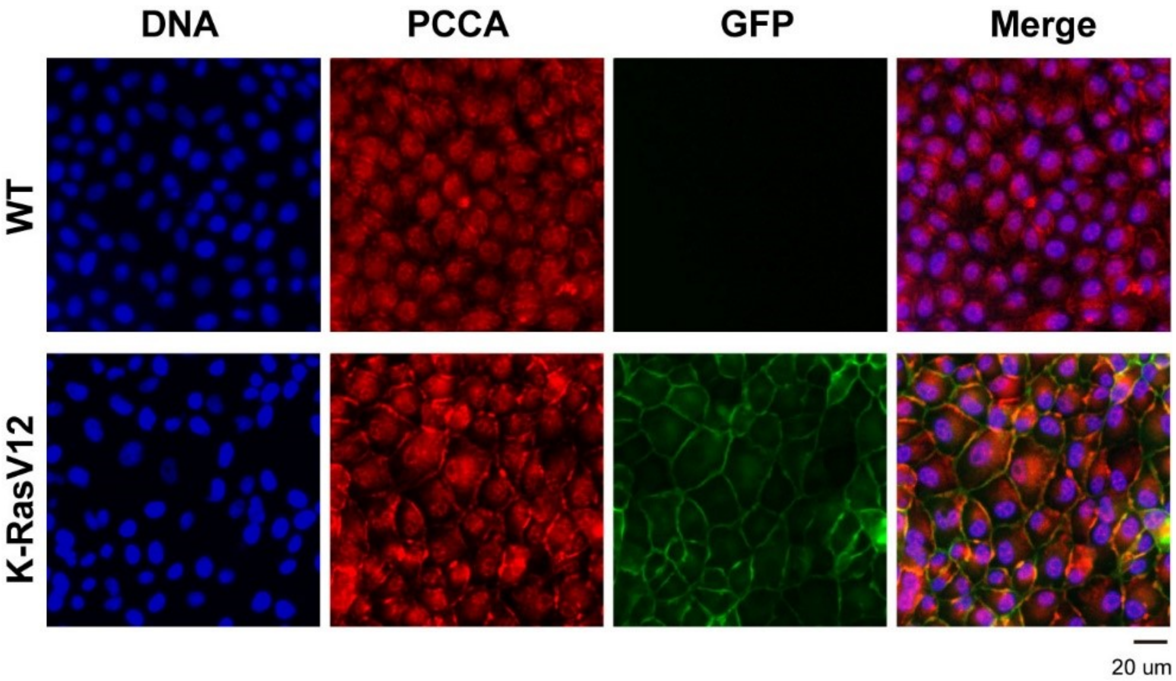

Figure S11. Immunofluorescent analysis for localization of PCCA in permeabilized sample.
WT and K-RasV12 cells fixed with 4% PFA were stained using polyclonal anti-PCCA antibody after permeabilized treatment (0.1% triton X-100 at room temperature for 10 min). Representative images from two independent experiments are shown.

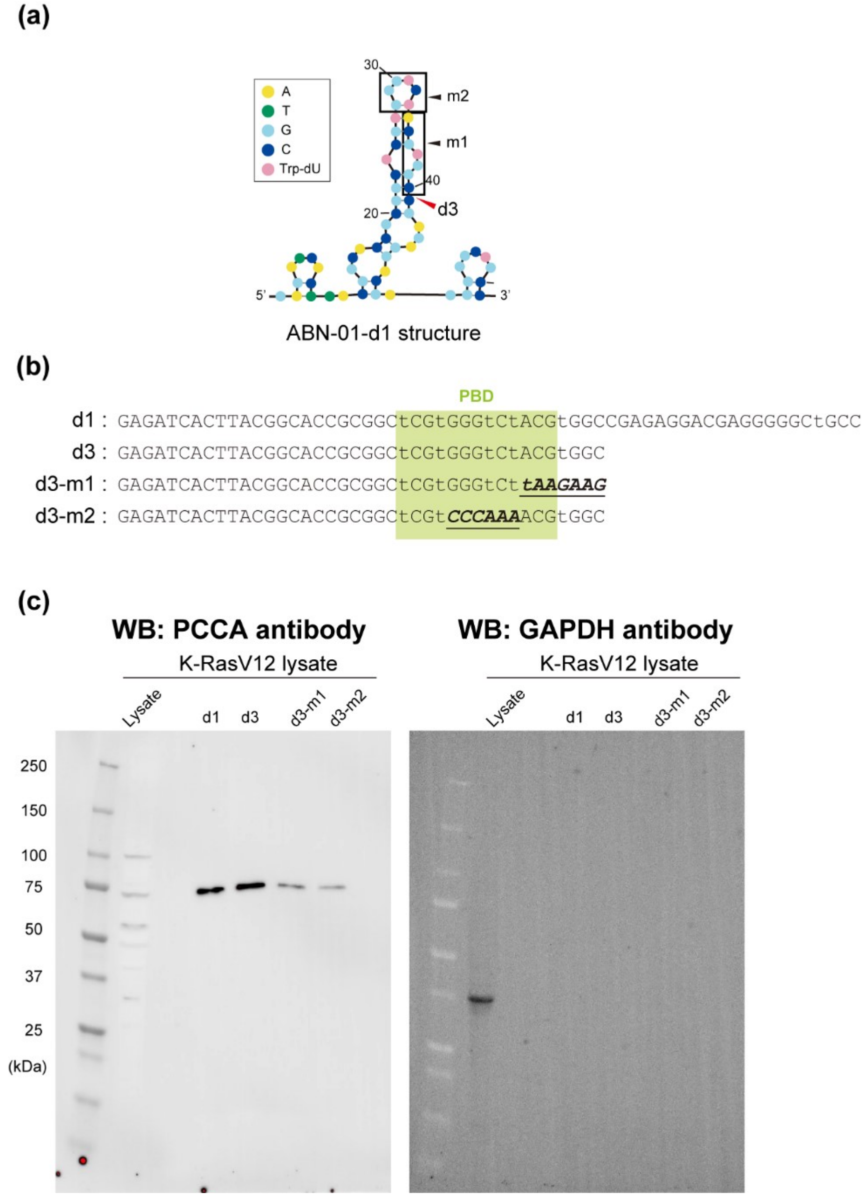

Figure S12. Domain analysis of the ABN-01 aptamer.
(a) Predicted secondary structure diagram of the ABN-01 aptamer is shown with mutation points. (b) Sequences of ABN-01-d1, -d3, -d3-m1, and -d3-m2 are shown. "t" letter means Trp-dU incorporation site. (c) Binding activity of mutant ABN-01-d3 was analyzed by aptamer-based precipitation followed by WB analysis. K-RasV12 lysate in the left lane was used for lysate control. Representative result from two independent experiments is shown.

Name	Sequence
Biotinylated library template	/5Biosg/CGCTGACCCGTGCCTTGG (N)43 GGTGCCGTAAGTGATCTCCC
Forward primer	GGGAGATCACTTACGGCACC
Biotinylated reverse primer	/5Biosg/CGCTGACCCGTGCCTTGG
TYE665-labeled forward primer	/5TYE665/TT GGGAGATCACTTACGGCACC
Biotinylated forward primer	/5Biosg/GGGAGATCACTTACGGCACC
ABN-01 template	/5Biosg/CTGACCCGTGCCTTGGGGCAGCCCCCTCGTCCTCTCGGCCACGTAGACCCACGAGCCGCGGTGCCGTAAGTGATCTC
ABN-02 template	/5Biosg/CTGACCCGTGCCTTGGTCCGGCGTAGACCCACGACGTCCCAGCCCGTCCTCGCTCCTTCGGTGCCGTAAGTGATCTC
ABN-03 template	/5Biosg/CTGACCCGTGCCTTGGGGATTCTCGTAGACCCACGATCCCCCCTCTCGCGTAGTGGCCGGGTGCCGTAAGTGATCTC
ABN-04 template	/5Biosg/CTGACCCGTGCCTTGGTCGGTCTCGTCGTAGACCCACGACTCCTGTCCGCTCACCTTTCGGTGCCGTAAGTGATCTC
ABN-05 template	/5Biosg/CTGACCCGTGCCTTGGGCTTACTCCTCGGGTCCCGCCGCTCGTAGAACCACGATCGGTCGGTGCCGTAAGTGATCTC
ABN-06 template	/5Biosg/CTGACCCGTGCCTTGGCCTCCAGCAACTCGTAGACCCACGAGTGGTACCCGCCCCCCGGGGTGCCGTAAGTGATCTC
ABN-07 template	/5Biosg/CTGACCCGTGCCTTGGAGGATGACTCCGCCCCTCCCCGTCGTAGATCCACGACGCGTCCGGTGCCGTAAGTGATCTC
ABN-08 template	/5Biosg/CTGACCCGTGCCTTGGGGTGCGCCTGATGCCCCTTCTCTGAGCTGTTTGTCCGCGCCGGGGTGCCGTAAGTGATCTC
ABN-09 template	/5Biosg/CTGACCCGTGCCTTGGTGTCTCGTAGACCCACGAGAGGGCCTCGCTGGCCGGCGACCGGGGTGCCGTAAGTGATCTC
ABN-10 template	/5Biosg/CTGACCCGTGCCTTGGCACGCCGCGCGCGGGGGTTTGCCGACGCAGTCCTGGGCTTGGCGGTGCCGTAAGTGATCTC
ABN-01-d1 template	/5Biosg/GGCAGCCCCCTCGTCCTCTCGGCCACGTAGACCCACGAGCCGCGGTGCCGTAAGTGATCTC
ABN-01-d2 template	/5Biosg/CCTCGTCCTCTCGGCCACGTAGACCCACGAGCCGCGGTGCCGTAAGTGATCTC
ABN-01-d3 template	/5Biosg/GCCACGTAGACCCACGAGCCGCGGTGCCGTAAGTGATCTC
ABN-01-d4 template	/5Biosg/ACGTAGACCCACGAGCCGCGGTGCCGTAAGTGATCTC
ABN-01 templete for pull-down	CTGACCCGTGCCTTGGGGCAGCCCCCTCGTCCTCTCGGCCACGTAGACCCACGAGCCGCGGTGCCGTAAGTGATCTC
Control aptamer template for pull-down	CTGACCCGTGCCTTGGGCTCGGCTTGCCTTAGTGGGTCTCGACCCCAAAAGGTCTCGTAGGTGCCGTAAGTGATCTC
oligo 1 (equal mixture of A&B)	
A*	GTCAATCGATCGTATCAGTCCAC (N)15 GAG
B*	GTCAATCGATCGTATCAGTCCACTTTTTCCTTTTTAATTTTTGAG
oligo 2	CTGTCAATCGATCGTATCAGTCCACAA (N)43 GCATGACTCGAACGGATTAGTGACTAC
Forward with adaptor sequence	TCGTCGGCAGCGTCAGATGTGTATAAGAGACAGGGGAGATCACTTACGGCACC
Reverse with adaptor sequence	GTCTCGTGGGCTCGGAGATGTGTATAAGAGACAGCGCTGACCCGTGCCTTGG
Forward ACTB primer	GGCACCCAGCACAATGAAG
Reverse ACTB primer	ACAGTGAGGCCAGGATGGAG
Forward PCCA primer	TGTAGCTCTTGCCAAAGCAG
Reverse PCCA primer	ACTAGGTCCAGGCCGGTAAT

*Trp-dUTP-included oligo at position T or N

Table S1. Oligonucleotides used in this study.

	Round 1	Round 2	Round 3	Round 4	Round 5
Library concentration	100 nM	50 nM	40 nM	25 nM	12.5 nM
Dish type	12-well	24-well	24-well	24-well	24-well
Volume	500 uL	300 uL	300 uL	300 uL	300 uL
Blocking : oligo 1	–	–	25 nM	50 nM	65 nM
Blocking : oligo 2	1000 nM	1000 nM	2000 nM	5000 nM	5000 nM
Competitor : oligo 1	–	–	100 nM	100 nM	110 nM
Competitor : oligo 2	–	–	–	1000 nM	3000 nM
Binding time (pre-clear)	–	10 min	10 min	10 min	10 min
Binding time	30 min	30 min	30 min	30 min	30 min
Selection temperature	on ice	on ice	25 ºC	25 ºC	25 ºC
2-min washes	–	1	2	3	3

Table S2. Cell-SELEX conditions used in this study.

SUPPLEMENTARY REFERENCE

27. A. Ohoka, M. Kajita, J. Ikenouchi, Y. Yako, S. Kitamoto, S. Kon, M. Ikegawa, T. Shimada, S. Ishikawa and Y. Fujita, *J Cell Sci*, 2015, **128**, 781-789.
